# Quinoa bioester application shifts human skin proteome toward molecular profiles associated with younger age

**DOI:** 10.1038/s42003-026-10006-4

**Published:** 2026-04-09

**Authors:** Amanda C. Camillo-Andrade, Lucas A. Sales, Carolina M. Catarino, Bruna Bosquetti, Camila F. S. Oliveira, Patricia H. Szuchman, Andrezza D. P. M. Canavez, Ana Raquel I. Firmino, Rodrigo C. Romanhole, Gustavo Dieamant, Desiree C. Shuck, Rosario Duran, Juliana S. G. Fischer, Marlon D. M. Santos, Paulo C. Carvalho

**Affiliations:** 1 Laboratory for Structural and Computational Proteomics, Carlos Chagas Institute, Fiocruz, Paraná Brazil; 2 Analytical Biochemistry and Proteomics Unit, Instituto de Investigaciones Biológicas Clemente Estable, Institut Pasteur de Montevideo, Montevideo, Uruguay; 3 Positivo University, Paraná, Brazil; 4 Product Performance Department, Grupo Boticário, Curitiba, PR Brazil; 5 Research and Development Department, Grupo Boticário, Curitiba, PR Brazil; 6 Integrated Space Stem Cell Orbital Research (ISSCOR) Center, University of California, San Diego, CA USA

**Keywords:** Biochemistry, Computational biology and bioinformatics

## Abstract

Skin aging involves complex molecular changes that current strategies struggle to reverse. Here, we developed a machine learning approach using Support Vector Regression to predict biological skin age from proteomic profiles, enabling objective assessment of anti-aging formulations. In this exploratory, proof-of-concept, prospective observational study, we set out to characterize treatment-induced proteomic changes in human skin as the pre-specified primary outcome. Women (ages 20–80) received 30-day topical quinoa bioester application on one forearm, with the contralateral forearm receiving vehicle. Mass spectrometry revealed significant upregulation of barrier function proteins (desmoglein-1, filaggrin), antioxidant enzymes (SOD1, glutaredoxin-1), and protease inhibitors. Our Support Vector Regression (SVR) model, trained on pre-treatment proteomes, predicted lower proteomic ages for quinoa bioester-treated skin compared to vehicle-treated skin, with observed median differences of 11 and 16 years for participants under and over 50, respectively (p < 0.01 for participants ≥50 years). While these values do not necessarily correspond to biological years, these findings demonstrate that topical bioactives can induce detectable shifts in skin proteomic profiles. These results establish a quantitative framework for evaluating skin rejuvenation strategies and suggest quinoa bioester as a promising anti-aging cosmeceutical.

## Abstract (web summary)

Paired skin proteomic comparisons within the same individuals reveal that quinoa bioester application modulates barrier and antioxidant proteins, shifting molecular profiles toward those associated with a younger age, with pronounced effects after age 50.

## Introduction

The aging process underlies a progressive decline in organ function, including the skin, which serves as the primary protective barrier against environmental stressors such as ultraviolet (UV) radiation, pollution, microbial pathogens, and chemical agents^1,2^. In recent years, the concept of the skin exposome has emerged, recognizing that both intrinsic and extrinsic factors interact in a complex manner to influence the biological and clinical signs of skin aging^3^*.* With increasing life expectancy, there is a growing demand for effective strategies to maintain skin health. Bioactives like quinoa bioester stand out for their potential, offering benefits compared to other natural compounds such as polyphenols and peptides^4,5^. Intrinsic aging, driven by genetic and metabolic factors, results in a progressive decline in skin structure and function^6^. This includes decreased fibroblast activity, reduced collagen synthesis, and elastin degradation, leading to less elasticity, increased laxity, and the formation of wrinkles. Additionally, intrinsic aging slows cellular turnover and reduces moisture retention, impairing the skin’s barrier function and repair mechanisms^7^.

Extrinsic factors intensify these degenerative changes. UV radiation, in particular, induces the production of reactive oxygen species (ROS), activates matrix metalloproteinases (MMPs), and promotes the degradation of extracellular matrix (ECM) components, further destabilizing dermal architecture^7^. Consequently, natural degeneration is accelerated by extrinsic factors, justifying more effective, targeted therapeutic strategies to address the multifactorial nature of skin aging. To better understand the complex biological processes driving these changes, researchers are increasingly leveraging omics techniques—such as genomics, transcriptomics, and proteomics—to comprehensively profile the molecular alterations associated with aging^4,8,9^.

In response to this need, bioactive compounds derived from natural sources have gained increasing attention for their potential in cosmeceutical applications aimed at mitigating skin aging and improving skin health^5,10^. These bioactives, including polyphenols, peptides, and fatty acids, exhibit pharmacological properties such as antioxidant, anti-inflammatory, and anti-matrix metalloproteinase activities, which directly counteract the molecular mechanisms involved in skin aging^11^. Among these, the quinoa bioester derived from *Chenopodium quinoa* has been identified for its involved role in promoting skin replenishing^4^. Rich in essential fatty acids and phenolic compounds, quinoa bioester has demonstrated the ability to modulate keratinocyte differentiation, enhance epidermal barrier function, inhibit collagenase activity, and protect against oxidative stress. These properties underscore its potential for alterations at the molecular level^4,12^.

Proteomics stands as a powerful tool for dermatological research as it can provide a comprehensive elucidation of the molecular pathways and signatures involved in skin aging. In this study, we leverage proteomic analysis to evaluate the effects of quinoa bioester across different skin ages^13^. Nevertheless, proteomic studies in humans present challenges due to inherent biological variability, influenced by genetic diversity, environmental exposures, and inter-individual differences^14^. Moreover, interpreting alterations in the proteome and linking them to specific biological processes, such as aging, is not straightforward. Discussions based on traditional approaches that focus only on differentially abundant proteins lists may introduce bias by having the researcher selectively emphasizing proteins that fit preconceived notions of aging-related changes.

Here, we employed a differential proteomic pipeline using paired sampling from the forearms of 61 individuals to better address the challenge of inter-individual variability through a refined statistical approach, enhancing sensitivity. To achieve this, data analysis was performed using the Pairwise Comparer module^15^ from PatternLab for proteomics^16^. Collecting pre- and post-treatment samples from the same forearm of each participant ensured a better-controlled environment, enhancing consistency and reliability in the analysis.

We also present an unbiased approach for evaluating treatment effectiveness by leveraging skin proteomic profiles to develop a molecular age predictor. This was achieved using a Support Vector Regression (SVR) trained on proteomic data from individuals across a wide age range. SVR effectively handles high-dimensional data and is less prone to overfitting through regularization^17^, a strategy that penalizes overly complex models by adding a penalty for attempting to fit every detail in the data, which could lead it to perform poorly on new, unseen data. Additionally, using SVR to predict a proteomic age as a surrogate for treatment effectiveness avoids biases that may occur when researchers selectively choose to argue in favor of a particular product outcome.

## Results and discussion

### Bilateral forearm sampling and systematic product allocation to minimize experimental bias and enable paired analysis

**Fig. 1:**
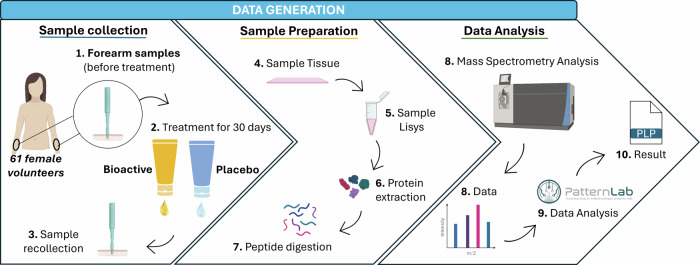
Data generation workflow for skin proteomic analysis. The diagram outlines the workflow for generating data in the proteomic analysis of skin samples. The process begins with the collection of samples from 61 female volunteers, where skin samples from the forearm are obtained before treatment. After a 30-day treatment with either a bioactive formulation or a vehicle, a second collection is performed. The samples then undergo preparation, including tissue processing, cell lysis, protein extraction, and enzymatic digestion into peptides. Mass spectrometry analysis is then performed using an Orbitrap Lumos, and protein identification and quantitation performed using the PatternLab for proteomics software. Image created using Microsoft PowerPoint, incorporating elements from BioRender. Sales, L. (2026) https://BioRender.com/l35kb1o.

Aiming toward establishing a well-controlled evaluation of quinoa bioester effects, we employed a bilateral forearm sampling strategy with systematic balanced treatment allocation. Each of the 61 participants provided four skin samples—one from each forearm—before and after a 30-day treatment period, resulting in a total of 244 samples. One forearm was treated with the quinoa bioester formulation, while the contralateral forearm received a vehicle. Our motivation in alternating treatment assignment between forearms was to help minimize potential biases introduced by lateral dominance, ensuring that any observed differences could be attributed to the treatment rather than physiological asymmetries. Another motivation of this design was to enable a paired analysis approach, enabling within-subject comparisons and the use of paired statistical methods to better account for inter-individual variability (Fig. 1).

### Large-scale mass spectrometry identifies thousands of skin proteins

All samples underwent proteomic analysis using mass spectrometry in technical duplicates, yielding a total of 488 raw files. A total of 45,697,082 tandem mass spectra were acquired, of which 5,100,014 yielded confident identifications, mapping to 40,592 peptides and up to 2906 protein sequences. A quality control assessment of these data as provided by RawVegetable^18,19^ is available in Supplementary data 2. To the best of our knowledge, this is the largest proteomic dataset of skin produced to date. Identifications, as per PatternLab for Proteomics V, are available in the Supplementary data 3.

### Paired proteomic analysis reveals that quinoa bioester modulates proteins linked to skin integrity, oxidative defense, and regeneration

Paired proteomic studies can provide a significant advantage as they allow within-subject comparisons, mitigating the impact of inter-individual variability and enhancing statistical power and reliability. The comparison of paired samples from the same individual allows us to better account for intrinsic biological differences, ensuring that the observed proteomic alterations were primarily due to environmental exposure rather than unrelated individual variability^15^.

Our paired proteomic analysis revealed that quinoa bioester treatment resulted in significant alterations (*p* < 0.05) in the abundance of proteins associated with skin structure, antioxidant defense, protease activity, and cellular homeostasis. Below, we highlight key proteins with differential abundancy in quinoa bioester-treated samples compared to the vehicle-treated forearm skin over a 30-day period. All proteins discussed in this section were identified in both forearms of at least 30 people. A complete list of differentially abundant proteins, including Log_2_ fold changes and *p*-values identified in the paired analysis between quinoa bioester and vehicle treatments, is available in Supplementary data 4. The first set of proteins is related to maintaining the skin’s barrier function and structural cohesion. Desmoglein-1 (DSG1, Q02413) and desmocollin-1 (DSC1, Q08554), are components of desmosomes that facilitate intercellular adhesion in the epidermis, strengthen keratinocyte cohesion, enhancing mechanical resilience and counteracting age-related loosening of skin structure^20^. Their increased in abundance in quinoa bioester-treated samples could lead to improved skin elasticity and firmness, characteristics often diminished with aging. Likewise, filaggrin (FLG, P20930), a key protein in keratinocyte differentiation and formation of the stratum corneum, is linked with improvements in the skin barrier function and hydration^21^ by generating natural moisturizing factors during its degradation^22^.

Oxidative stress is a major contributor to skin aging, primarily through the generation of reactive oxygen species (ROS) that damage cellular components^23^. Our data showed an increase in the abundance of antioxidant enzymes such as superoxide dismutase [Cu-Zn] (SOD1, P00441), glutaredoxin-1 (GLRX, P35754), and glutathione synthetase (GSS, P48637) in the quinoa bioester-treated forearms. SOD1 catalyzes the dismutation of superoxide radicals into hydrogen peroxide and oxygen, mitigating oxidative damage^24^. Glutaredoxin-1 and glutathione synthetase play roles in maintaining the redox state of proteins and synthesizing glutathione, a major cellular antioxidant^25^. In contrast with these proteins, S100-A14 is downregulated in the quinoa bioester-treated samples, indicating that, compared to the vehicle, the quinoa bioester-treated skin experiences reduced oxidative stress, likely due to the bioactive’s role in aiding repair mechanisms^26^. Notably, this contrasts with our previous findings, where S100-A14 was upregulated in chronically sun-exposed facial skin, indicating increased oxidative stress in those areas^15^.

Serine protease inhibitors such as serpin B3 (SERPINB3, P29508), serpin B7 (SERPINB7, O75635), and serpin A12 (SERPINA12, Q8IW75) had increased abundancy in the quinoa bioester-treated samples. This suggests an enhanced regulation of proteolytic activity, facilitating skin renewal while preventing excessive degradation of structural proteins. Notably, serpin B7 plays a crucial role in enhancing the skin’s barrier function by promoting keratinocyte differentiation. Maintaining this balance between proteases and their inhibitors is essential for skin homeostasis and for preventing the breakdown of vital extracellular matrix components such as collagen and elastin^27–29^.

Finally, we spotlight the abundance of dermcidin (DCD, P81605), an antimicrobial peptide typically produced by sweat glands, linked with a strengthening of the skin’s innate immune defenses. The elevated levels observed in treated samples suggest an enhanced protective response, potentially reducing the risk of skin infections that can exacerbate inflammatory processes and accelerate aging^30–32^.

To complement our proteomic findings, we assessed skin hydration changes using bioelectrical impedance measurements. Hydration analysis across all 61 participants revealed that both the vehicle and the quinoa bioester treatment produced improvements in skin hydration (mean Δ = 0.54 arbitrary units for vehicle vs 1.14 for bioactive, *p* = 0.17), with the quinoa bioester group showing improvement compared to vehicle. While this difference did not reach conventional statistical significance (*p* > 0.05), the consistent directional improvement suggests a meaningful biological effect. The high inter-individual variability expected given our diverse age range (20–80 years), combined with the hydrating effects of the vehicle base formulation itself, makes detecting additional bioactive-specific effects particularly challenging through conventional metrics alone. Skin hydration measurement data are available in Supplementary data 5.

### Support Vector Regression (SVR) identifies proteomic age shifts associated with quinoa bioester treatment

In our study, we employed SVR to assess whether it is possible to predict skin age based on its proteomic profile. If so, could this prediction-model then detect proteomic age shifts following quinoa bioester formulation?

### SVR-based pipeline for proteomic age prediction

In brief, SVR^17^ is a machine learning technique rooted in the principles of Support Vector Machines (SVM)^33^ but, unlike SVM, which is designed for classification, SVR is specifically tailored for regression tasks. Both methods are widely used due to their ability to handle high-dimensional data efficiently, meaning datasets with a large number of features, where each protein’s quantitation value represents a distinct dimension. Unlike conventional regression approaches that minimize the error between predicted and actual values directly, SVR seeks to balance empirical error, the deviation observed in the training dataset, with a theoretical generalization error, which represents the model’s expected performance on unseen data.

A key feature of SVR is its use of kernels, which transform input data into a higher-dimensional space where complex relationships can be more easily captured. In simple terms, a kernel is a mathematical function that allows SVR to learn patterns in data that are not linearly separable in their original form. While nonlinear kernels, such as polynomials, can model intricate relationships, they also increase the risk of overfitting, where the model becomes excessively tailored to the training data, capturing noise rather than meaningful biological trends—especially when working with limited datasets. Overfitting leads to poor generalization, meaning the model performs well on training data but fails to predict new, unseen samples accurately. Given the high dimensionality of our proteomic dataset and the need for interpretability, we opted for the simplest kernel function, i.e., the linear kernel, inspired on Occam’s razor—favoring the simplest model that adequately explains the data. By assuming a direct relationship between protein abundance and biological age, the linear kernel allows us to assess individual protein contributions while maintaining a balance between complexity and generalizability. This decision is supported by the success of linear models in established molecular aging clocks, including the epigenetic clocks by Horvath^34^ and Hannum et al.^35^, both of which employed elastic net regression, a penalized linear approach, to accurately predict age from high-dimensional methylation data.

**Fig. 2:**
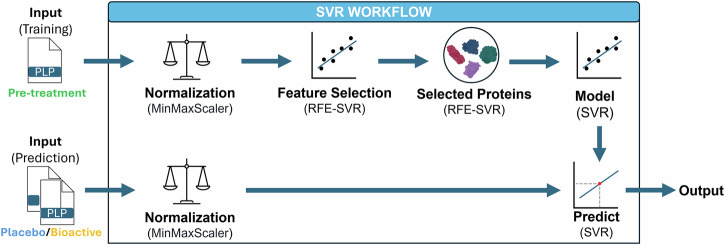
The diagram illustrates the SVR-based workflow for proteomic age prediction. The model is trained on shotgun proteomic data from pre-treated forearms samples, undergoing recursive feature elimination (RFE) in conjunction with the SVR to select a subset of proteins that are both predictive of age and (hopefully) biologically informative. The trained model is then applied to predict the proteomics ages of vehicle- and bioactive-treated forearms. All data undergoes normalization using the MinMaxScaler, and only proteins selected by RFE-SVR are used for prediction. Image created using Microsoft PowerPoint, incorporating elements from BioRender. Sales, L. (2026) https://BioRender.com/l35kb1o.

To apply SVR for age prediction, we designed a computational pipeline that systematically processes proteomic data, ensuring the selection of the most informative protein features while minimizing biases. The first step involves normalizing protein quantitation values to bring them to a comparable scale, which is essential for stable model training. Next, we employ Recursive Feature Elimination (RFE) in conjunction with SVR to identify a subset of proteins most predictive of biological age, removing those that contribute little to the model’s performance. The final trained model is then used to estimate the predict proteomic age of post-treatment samples, allowing us to assess whether quinoa bioester treatment leads to measurable shifts in the skin proteome. An overview of this workflow is depicted in Fig. 2.

### Evaluating the skin proteome’s age-predictive potential using leave-age-out cross-validation

Before applying SVR to detect age shifts induced by treatment, which is our ultimate goal, we first needed to assess whether proteomic data alone encodes sufficient biological information to predict chronological age—an essential step in determining the feasibility and potential sensitivity of this approach for detecting rejuvenation-associated trends. Given the complexity of skin aging and the inherent variability in proteomic datasets, identifying a clear molecular signature of age is a challenging task. If a discernible trend exists, even within the limitations of our dataset, this step would be a decisive factor in deciding if we could continue with our investigations, in using SVR as a tool for assessing rejuvenation-associated effects, and even for justifying future studies with larger cohorts.

**Fig. 3:**
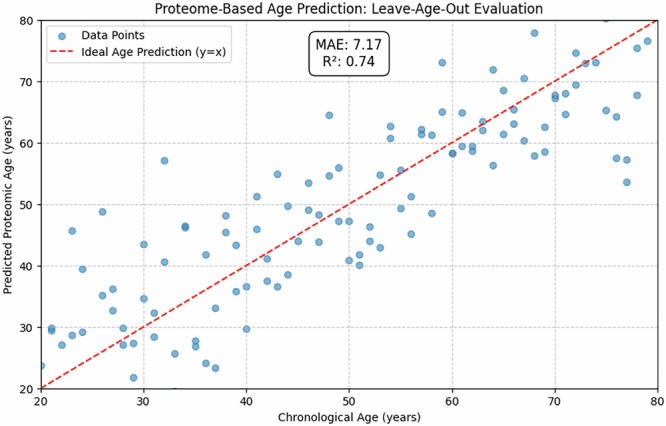
Leave-age-out validation demonstrates the predictive power of skin proteome for estimating biological age. This scatter plot illustrates the ability of a support vector regression (SVR) model trained on skin proteomic data to predict chronological age. The model was evaluated using a Leave-Age-Out (LAO) approach, where all data from individuals, *n* = 61 individuals (one per age from 20 to 80), of a given age were systematically left out during training and subsequently used for independent prediction. Each point represents an individual’s chronological age (*x*-axis) plotted against the predicted proteomic age (*y*-axis), The red dashed line (*y* = *x*) represents what would be the result of a perfect prediction, where predicted and actual ages align exactly. The model achieved a mean absolute error (MAE) of 7.17 years and an R² value of 0.74, indicating a correlation between proteomic features and chronological age.

To achieve this, we evaluated our SVR model by using what we referred to as “Leave-Age-Out” cross validation (LAO-CV), which is essentially our playful way of referring to the well-known “Leave-One-Out” cross-validation method, emphasizing our specific focus on age. The LAO-CV iteratively excludes all samples corresponding to a specific age, ensuring the model is trained on all the remaining data and tested on the left-out age. Figure 3 show our results for the LAO-CV and the plot show a trend when comparing the predicted proteomic age with the chronological age.

While this section alone does not constitute definitive evidence of a predictive model, the observed trend provided us with sufficient motivation to proceed with our investigation. This step was crucial as it offered insight into the variability of our model within the untreated dataset and suggested that the skin proteome likely encodes sufficient biological information to infer an individual’s age—a capability that, to our knowledge, has not been previously demonstrated. A rigorous validation would require an independent dataset; however, at this stage, given that we collected samples from the same individuals one month later following vehicle and quinoa bioester treatment, these post-treatment datasets can serve as a practical test for model generalization. If age predictions remain consistent, to some extent in this new dataset, it would serve as a critical validation step for this stage of our study, reinforcing the rationale for applying SVR to assess rejuvenation-associated effects.

An important consideration in our study design is the use of samples from the same individuals for both model training (pre-treatment) and testing (post-treatment). Such experimental design is motivated for detecting within-subject treatment effects rather than building a generalizable age predictor for new populations. The massive inter-individual variability in human skin proteomics, which far exceeds treatment-induced changes, necessitates paired analysis to better focus on treatment effects. We emphasize that our SVR model serves as an analytical tool for quantifying predict proteomic age shifts within individuals, and not as a deployable age prediction system. Future studies seeking to develop generalizable proteomic age clocks would require independent and unpaired validation cohorts.

### Quinoa bioester treatment lowers predicted proteomic age

**Fig. 4:**
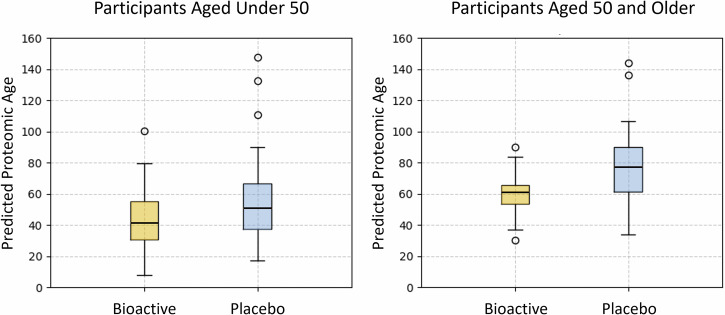
Predicted proteomic age of quinoa bioester- and vehicle-treated skin, stratified by participant age group. The left panel represents participants aged under 50 (*n* = 30), while the right panel represents participants aged 50 and older (*n* = 31). Boxplots compare the predicted proteomic ages (derived from the SVR model trained on pre-treatment proteomic profiles) for bioactive-treated versus vehicle-treated forearms from the same individuals after 30 days of treatment. In each boxplot, the center line represents the median, box edges represent the first and third quartiles (Q1 and Q3), and whiskers extend to the most extreme data points within 1.5 times the interquartile range (IQR) from the box edges. Points beyond the whiskers represent outliers. Statistical significance was assessed using the Wilcoxon signed-rank test for paired comparisons: “*p* = 0.14 for the <50 group (observed median difference of 11 years) and *p* < 0.01 for the ≥50 group (observed median difference of 16 years), comparing bioactive to vehicle within each age stratum. These values demonstrate differential treatment effects and do not necessarily correspond to biological years. The differences between age groups reflect the expected age-related changes in skin proteome, with older participants showing higher predicted proteomic ages in both treatment conditions.

The predictive model trained on pre-treatment samples was then applied to estimate the proteomic age of the same individuals after one month of treatment with either the vehicle or quinoa bioester formulation. It is important to note that the vehicle formulation itself contains a base that promotes skin hydration and, consequently, improves skin health, making the detection of additional bioactive-driven effects more challenging. Furthermore, the act of applying a topical formulation for a month inherently increases skin hydration, an important factor in maintaining skin integrity and function. Given these considerations, distinguishing the specific impact of the quinoa bioester from general improvements associated with vehicle treatment requires careful analysis. Additionally, since menopause is known to induce significant changes in skin structure and composition, typically occurring around the age of 50, we stratified our analysis into two groups: participants under 50 and those 50 years and older. However, it is important to clarify that we did not train separate models for each group. Instead, we applied the same SVR model trained on all pre-treatment samples—spanning the full age range—which had previously demonstrated its predictive capacity in the Leave-Age-Out analysis (Fig. 3). This ensures that any observed differences in predicted proteomic age reflect treatment effects rather than age-specific model biases. The results of these age-stratified predictions are presented in Fig. 4.

Figure 4 suggests that our age prediction model, previously trained only on the untreated samples, successfully captured age-related differences of this unseen dataset. In both treatment conditions, predicted ages were consistently higher for the older cohort ( ≥ 50 years) compared to the younger cohort: the quinoa bioester-treated samples showed median ages of 61 years (Q1-Q3: 53–66) versus 42 years (Q1-Q3: 31–55), while vehicle -treated samples showed median ages of 77 years (Q1-Q3: 61–90) versus 51 years (Q1-Q3: 37–66). In conclusion, the age-related differences were statistically significant when comparing the younger and the older group (Mann-Whitney U test, *p* < 0.01 for both bioactive and vehicle groups).

With this validation established, we examined the effects of quinoa bioester treatment on predicted proteomic age. The magnitude of the differential treatment effect showed an age dependence. Younger participants showed an observed median difference of 11 years between treatments (*p* = 0.14, Wilcoxon signed-rank test), while older participants showed an observed median difference of 16 years (*p* < 0.01, Wilcoxon signed-rank test). These numerical differences do not necessarily reflect biological years due to inherent limitations of applying the model to post-treatment data (domain shift), but indicate a differential effect that is more pronounced in older skin. This enhanced effect in older participants is especially noteworthy given that skin aging typically accelerates post-menopause, suggesting that quinoa bioester could be particularly beneficial for this group.

The consistency of these findings across both age cohorts, coupled with the more pronounced effects in older participants, supports a directional proteomic effect of quinoa bioester relative to vehicle. Notably, quinoa bioester treatment reduced outcome variability by 37% in younger participants (SD: 18.9) and 47% in older participants (SD: 13.1), suggesting it may help establish a more youthful and stable proteomic profile, regardless of the participant’s chronological age. The proteomic age difference observed between quinoa bioester and vehicle treatments was supported by quantitative hydration measurements, which showed a 111% greater improvement compared to vehicle. This multi-level evidence—combining molecular signatures with functional skin parameters—corroborates to our conclusion that quinoa bioester provides measurable skin health benefits.

The observed reduction in predicted proteomic age following quinoa bioester treatment aligns with its known biological properties and suggests broader cosmeceutical potential. The SVR model’s ability to detect these age-related shifts likely stems from quinoa bioester’s multi-target action on key aging pathways. Rich in essential fatty acids (particularly linoleic and oleic acids) and phenolic compounds, quinoa bioester directly addresses multiple hallmarks of skin aging. The proteins driving the SVR age predictions, including those involved in barrier function (DSG1, DSC1, FLG), oxidative defense (SOD1, GLRX, GSS), and protease regulation (SERPINB3, SERPINB7), represent convergent pathways that quinoa bioester modulates simultaneously. This coordinated action on multiple aging mechanisms, rather than targeting a single pathway, may explain the pronounced differential effect between treatments observed in our model.

Our topical treatment results suggest potential for both preventive and cosmeceutical potential. The more pronounced effects in older participants (≥50 years) indicate that quinoa bioester may be particularly effective in addressing accumulated age-related damage, possibly through its ability to restore compromised barrier function and reduce oxidative stress; both of which intensify with age. The 30-day treatment period demonstrated measurable proteomic shifts, suggesting that relatively short-term cosmeceutical applications can achieve molecular-level rejuvenation. Future applications could explore optimized delivery systems, combination strategies with other bioactives, or systemic formulations to extend these benefits beyond the treated skin areas. The fact that these changes are detectable through an unbiased machine learning approach strengthens the biological relevance of quinoa bioester as an anti-aging cosmeceutical with measurable molecular effects. For comprehensive pathway enrichment analyses of quinoa bioester’s mechanisms, we refer readers to our previous work in controlled reconstructed human epidermis (**Camillo-Andrade et al., 2020**), as the current in vivo study prioritized validating these effects in real-world human skin through our unbiased SVR approach.

To contextualize our proteomic findings, it is important to consider established benchmarks in anti-aging research, while acknowledging that different methods of evaluating aging, such as clinical parameters, epigenetic clocks, and proteomic profiles, represent fundamentally different measurement approaches, akin to using different rulers that may not be directly comparable. That said, topical strategies have been reported to yield modest effects over weeks; for example, vitamin C formulations have shown ~10–15% improvements in skin-aging parameters^36^, and tretinoin, to enhance collagen remodeling^37^. Importantly, proteomic profiling of topical cosmeceutical effects has rarely been performed in humans; most studies have focused on pharmaceutical actives or irritants (^38,39^), with cosmeceutical ingredients typically evaluated only in cell cultures and 3D skin models^4^. Systemic strategies using epigenetic clocks demonstrate larger but still modest effects; for example, metformin shows approximately 2–3 years of biological age deceleration over extended periods^40^.

Our observed differences in predicted proteomic age between treatments must be interpreted with caution given the inherently noisy nature of proteomic data from human skin samples, which show a high inter-individual variability. We emphasize that these numbers represent a suggestive trend, and our primary contribution is demonstrating that machine learning can detect treatment-associated proteomic patterns in an unbiased way, that is, without relying on a specific marker such as collagen. In addition, the use of a paired design, where treated regions were compared against control regions within the same participant before analysis across individuals, helped reduce variability, since part of the biological ‘noise’ was normalized internally. Nevertheless, considering this caveat along with other limitations of the study, such as having only one participant per age, we recommend that our numerical predictions eventually be tested against larger and more diverse populations.

Several limitations of our study should be considered when interpreting these results. A key constraint is our sampling strategy of one individual per age, is enough to adequately capture the natural variability within each age group. Skin aging is a complex process influenced by numerous factors including genetics, lifestyle, and environmental exposures, making it unlikely that a single individual could fully represent the proteomic profile of their age group. In our case, we sampled women from the same city, with very similar skin traits. This limitation could be addressed in future studies by including multiple participants of the same age, and models for different skin phototypes and different regions of the world, as skin aging patterns can vary significantly across different ethnic backgrounds and geographical locations. While our technical replicates help control for analytical variability, biological replicates from multiple individuals of the same age would provide a more robust assessment of age-related proteomic changes and treatment effects. Despite these limitations, our study provides valuable initial insights into the potential of quinoa bioester for skin rejuvenation and establishes a framework for future, more comprehensive investigations.

This observational study presents an unbiased machine learning framework using Support Vector Regression to assess whether proteomic profiles can detect shifts in molecular signatures of skin following cosmeceutical application. By integrating paired proteomic analysis with machine learning, we demonstrate that quinoa bioester induces coordinated molecular changes in barrier function, oxidative defense, and protease regulation, providing a foundation for objective assessment of cosmeceutical effects and opening new avenues for evidence-based and unbiased evaluation of skin care formulations.

## Materials and methods

### Sample collection

This study was approved by the Research Ethics Committee of the Instituto Oswaldo Cruz, Fundacao Oswaldo Cruz (CEP FIOCRUZ/IOC; CAAE 38352020.8.0000.5248, Parecer 4.971.427). All ethical regulations relevant to human research participants were followed. Written informed consent was obtained from all participants. The original approved study protocol is provided in Supplementary Note 1. Our inclusion criteria considered only female individuals aged between 20 and 80 years with phototypes II to IV. Exclusion criteria encompassed conditions such as skin diseases, smoking, diabetes and pregnancy. Age-stratified enrollment. We prospectively used single-year quota sampling across the prespecified 20–80 range. Volunteers were openly recruited and screened with a standardized form, ID verification, and considering year of birth; we enrolled one participant per birth-year. Age was defined as completed years at baseline, ensuring one participant of each age.Skin samples were collected using a non-invasive, painless technique similar to microdermabrasion, developed by our team. Samples were taken from a standardized area on the volar (inner) aspect of both forearms, between the wrist and elbow crease, of 61 participants—each representing an age from 20 to 80 years (e.g., one participant aged 20, one aged 21, continuing up to 80). Following the initial sampling, participants were instructed to apply a topical formulation containing quinoa bioester on one forearm twice daily —in the morning and at night— and an identical vehicle topical formulation without quinoa bioester, on the other forearm for a minimum of 30 days. The treatment period of at least 30 days was chosen to balance feasibility and the expected timeframe for observing initial molecular changes^41^. Formulation allocation followed a systematic balanced rule: odd-aged participants received the quinoa bioester formulation on one forearm and the vehicle on the contralateral forearm, while even-aged participants received the reverse assignment. This ensured balanced distribution of the active formulation across left and right forearms. Formulation tubes were labeled ‘right forearm’ or ‘left forearm’ with accompanying written instructions to ensure correct application throughout the treatment period. Both formulations were identical in appearance and texture, and participants were blinded to which formulation contained the active ingredient. Skin hydration was quantitatively assessed using the SkinUp® digital analyzer, which measures bioelectrical impedance. This device has been validated against the gold-standard Corneometer® CM825^42^. Measurements were taken from the same standardized forearm areas before and after the 30-day treatment period. At the end of the treatment period, skin samples were recollected from the same regions for post-treatment analysis.

The volar forearm region was specifically chosen due to its relatively low exposure to ultraviolet radiation compared to other easily accessible body areas. Additionally, the physical limitation of using the same hand to apply the topical formulations on both forearms minimizes cross-contamination between treatments. These combined factors make the volar forearm an ideal anatomical site, reducing the impact of external variables. In total, our proteomic dataset comprised 122 samples before treatment and another 122 after, for a total of 244 samples, providing, as far as we know, the largest skin proteomic dataset.

### Sample preparation

Proteins from the skin samples were extracted using RapiGest SF Surfactant at a concentration of 0.1%, following the manufacturer’s instructions. For each sample, 100 micrograms of proteins were reduced with dithiothreitol (DTT) to a final concentration of 10 mM for 30 min at 60 °C. After cooling to room temperature, the samples were alkylated with iodoacetamide at a final concentration of 30 mM for 25 min in the dark at room temperature. The proteins were then digested with sequence grade modified trypsin at a ratio of 1/50 (E/S) for overnight at 37 °C. The enzymatic reaction was terminated by adding trifluoroacetic acid to a final concentration of 0.4% (v/v), followed by incubation for an additional 45 min to degrade the RapiGest SF Surfactant. The samples were then centrifuged at 18,000 × *g* for 10 min to remove any insoluble material. The resulting peptides were quantified using the Qubit 2.0 fluorometric assay (Invitrogen®), following the manufacturer’s instructions. Each sample was then desalted and concentrated using Stage-Tips^43^.

### Mass spectrometry

Peptides were analyzed using LC–MS/MS with an UltiMate 3000 system (Thermo Fisher®) connected online to an Orbitrap Fusion Lumos mass spectrometer (Thermo Fisher®). The peptide mixture was chromatographically separated on a column (15 cm in length, 75 μm I.D., C18-AQ 3.0 μm resin) at a flow rate of 250 nL/min, using a gradient from 5% to 40% ACN in 0.1% formic acid over 120 min. The mass spectrometer operated in data-dependent acquisition (DDA) mode, switching automatically between full scan MS and MS/MS with a dynamic exclusion of 30 s. Survey scans (200–2000 m/z) were acquired in the Orbitrap at a resolution of 60,000 at *m/z* 200. The most intense ions in a 2-s cycle were selected, excluding those unassigned and in a 1+ charge state, isolated and fragmented by higher-energy collisional dissociation (HCD) with normalized collision energy of 30. Fragment ions were analyzed at a resolution of 15,000 at 200 *m/z*. The mass spectrometer conditions included a 2.5 kV spray voltage, no sheath or auxiliary gas flow, a heated capillary temperature of 40 °C, predictive automatic gain control (AGC) enabled, and an S-lens RF level of 40%. Mass spectrometer scan functions and nLC solvent gradients were controlled using the Xcalibur 4.1 data system (Thermo Fisher®).

### Peptide spectrum matching (PSM)

Spectral Identification, peptide quantitation and differential proteomics was conducted using PatternLab for proteomics V software^16^, which is freely accessible at https://www.patternlabforproteomics.org. Sequences for *Homo sapiens* were downloaded from Swiss-Prot on July 7th, 2023. A target-decoy database was created to include both reversed sequences and 104 common mass spectrometry contaminants, excluding keratin, which was not considered a contaminant in this analysis. The Y.A.D.A. 3.0 deconvolution algorithm was used for preprocessing the data, allowing for multiplexed spectra identification^44^. The Comet search engine^45^ was employed for mass spectra identification, considering fully and semi-tryptic peptide candidates with masses ranging from 500 to 6000 Da, allowing for up to two missed cleavages, with a precursor mass tolerance of 35 ppm and MS/MS bins of 0.02 *m/z*. Only peptide candidates with six or more aminoacids were considered. The search parameters included carbamidomethylation of cysteine as a fixed modification and oxidation of methionine as a variable modification.

### Validation PSM

The validity of the PSMs was performed using the Search Engine Processor (SEPro)^46^. The identifications were categorized by charge state (2+ and ≥3 + ) and tryptic status, creating four distinct subgroups. For each subgroup, XCorr, DeltaCN, DeltaPPM, and Peaks Matches values were utilized to develop a Bayesian discriminator. Identifications were then sorted in nondecreasing order based on the discriminator score. A cutoff score was established to accept a false discovery rate (FDR) of 1% at the protein level, determined by the number of decoys^47^. This process was independently applied to each data subset, ensuring an FDR that was independent of charge state or tryptic status. After that, proteins with score below 2 or identifications deviating by more than 10 ppm from the theoretical mass were excluded. This final filter resulted in protein-level FDRs below 1% for all search results.

### Statistics and reproducibility

This study enrolled female participants (one per age, 20 to 80 years), each providing four skin samples (two forearms, before and after treatment), totaling 244 biological samples. All samples were analyzed by mass spectrometry in technical duplicates (488 raw files). Technical replicates were defined as repeated LC-MS/MS acquisitions of the same peptide preparation; biological replicates were defined as samples from different participants. Quality control and LC-MS-MS reproducibility of the mass spectrometry data was performed using RawVegetable^18,19^, and a detailed assessment is available in Supplementary data 2. No data were excluded from the analyses.

For differential proteomics, a paired one-sample t-test on log2 fold changes with Benjamini-Hochberg FDR correction (adjusted *p* < 0.05) was used, as detailed in the Paired Proteomic Analysis section. For comparisons of predicted proteomic age between treatments within the same individuals, the Wilcoxon signed-rank test was used. For comparisons between age groups, the Mann-Whitney U test was used. Skin hydration differences were assessed using a two-sided *t*-test. All reported *p*-values are two-sided.

### Paired proteomic analysis

We used the Pairwise-Comparer module in PatternLab for Proteomics V to compare the skin proteomes of quinoa bioester-treated and vehicle-treated forearms^15^. This module is specifically designed to account for the paired nature of the samples, by directly comparing the proteomic profiles from the same subject. This tool computes the protein fold changes between paired conditions by calculating the ratio peptide abundance in the treated versus vehicle samples for each individual. These log_2_(fold) changes are then subjected to a one-sample *t*-test, verifying against a null hypothesis mean of zero, which assumes no difference in protein expression between conditions. This process generates both fold changes and corresponding *p*-values, offering a comprehensive overview of the proteomic landscape.

Finally, a Benjamin-Hochberg false discovery rate (FDR) correction was applied to account for multiple comparisons, with proteins having an adjusted *p*-value below 0.05 considered significantly differentially expressed. Proteins showing statistically significant changes in abundance and having an absolute fold change above 30% as estimated by the average of the fold of the peptides, were considered differentially abundant and subsequently subjected to further biological interpretation.

**Leveraging Support Vector Regression (SVR) and Leave-Age-Out Cross Validation for Unbiased Predicted Proteomic Age from Skin Profiles**

### The tools and methodologies utilized to develop our pipeline

We aim to predict a proteomic age based on skin proteomic profiles. The SVR-based approach was later selected during the analytical phase as the most suitable method for evaluating proteomic age shifts, consistent with the exploratory nature of this study. For this, we used Python 3.8 with widely adopted libraries such as pandas for data handling, NumPy for numerical computations, scikit-learn for machine learning, and matplotlib for graphical visualizations. Our code is available at https://github.com/marlondms/AI-ON-SKIN/ and has been archived on Zenodo.

### Establishing a minimal key set of proteins using recursive feature elimination

To gain insights into the biological patterns driving skin aging and improve on the interpretability of our predictive model, we aimed to identify a minimal set of key proteins that retained strong predictive power for age estimation. Quantitative proteomic data from untreated forearms obtained using PatternLab for proteomics were exported to a CSV file. Protein quantitation values were normalized to a 0–1 range using MinMax scaling to ensure all features contributed equally to the model regardless of their original scale. The scaler was fitted exclusively on the pre-treatment training samples, and these same scaling parameters were subsequently applied to transform the post-treatment test samples, ensuring consistent normalization without incorporating any information from the test set distribution. All feature selection (RFE) were performed within a nested LAO framework; in each fold, feature selection and hyperparameter optimization were fit on the training ages only, then evaluated on the held-out age. We then employed Recursive Feature Elimination (RFE)^48^ in conjunction with a SVR model utilizing a linear kernel. The linear kernel was chosen due to its simplicity and interpretability, while reducing the risk of overfitting.

RFE is an iterative feature selection technique that ranks proteins based on their importance to the predictive model and systematically eliminates the least significant ones. The SVR model assigns weights to each protein, reflecting their contribution to age prediction. RFE uses these weights to iteratively remove proteins with the smallest values, thereby reducing the feature set while monitoring the impact on model performance. Initially, the SVR model was trained using the complete set of quantified proteins from our proteomic data, and after each iteration of the RFE, the Mean Absolute Error (MAE) was calculated to evaluate model performance. The MAE is the average magnitude of errors, calculated as the sum of the absolute differences between the predicted and actual ages, divided by the total number of samples. By doing this procedure, we systematically eliminated the least influential proteins and retrained the SVR model after each elimination, continuing this process iteratively. The MAE was recalculated at each step to assess how removing additional proteins affected predictive accuracy. The elimination process stopped once further removal of proteins led to a significant increase in the MAE, indicating a reduction in predictive power.

### Leave-age-out cross validation (LAO-CV)

We performed Leave-Age-Out cross-validation on pre-treatment samples (*n* = 122) to simultaneously optimize hyperparameters and evaluate model performance. Testing regularization parameter C values of [10, 100, 1000, 10000, 100000], we selected C = 100000 which achieved MAE = 7.17 years (R² = 0.74).

### Empirical evaluation of SVR model using training samples

We applied LAO-CV using our SVR model with optimized parameters and feature selection, excluding the age (one individual per age) being tested, on the full set of untreated forearm samples used for training. This evaluation is definitely not intended to serve as a definitive quality metric, as we acknowledge that assessing the model’s performance on the training data may inflate performance metrics due to overfitting. Our sole motivation at this step was to conduct an empirical assessment of whether the proteome could be used for inferring biological age, and whether our model could fit to our training dataset before applying it to treated samples. This preliminary check satisfied our curiosity by providing a measure of the empirical error before proceeding to the next step which is evaluating the model’s capacity to capture age-related patterns in the proteomic data of unseen samples.

### Assessing the effects of quinoa bioester treatment on proteomic age using SVR model

We trained an SVR model using all samples from the untreated group by considering the selected features and optimized hyperparameters. This model was then applied to infer the predict proteomic age of both the treated (i.e., vehicle and quinoa bioster groups) and untreated forearms for each subject. Note that these new treated samples were never “seen” by the model; by using the untreated data for training, we aimed to assess whether the treatment led to any observable shift in the proteomic profile that would reflect an improvement in “shift in the proteomic profile consistent with younger skin signatures”. The inferred ages were compared between the treated and untreated forearms to determine if the quinoa bioester and the vehicle treatment had a measurable impact on the ages inferred by our model.

### Reporting summary

Further information on research design is available in the Nature Portfolio Reporting Summary linked to this article.

## Supplementary information

Transparent Peer Review file

Supplementary Information

Description of Additional Supplementary Files

Supplementary Data 1

supplementary data 2

supplementary data 3

supplementary data 4

supplementary data 5

Reporting summary

## Data Availability

The mass spectrometry proteomics data have been deposited to the ProteomeXchange Consortium via the PRIDE^49^ partner repository with the dataset identifier PXD062216. Source data underlying the graphs in Figs. 3 and 4 are provided in Supplementary Data 1. Quality control assessments are available in Supplementary data 2, protein identifications in Supplementary data 3, differential proteomics results in Supplementary data 4, and skin hydration measurements in Supplementary data 5. All other data supporting the findings of this study are available from the corresponding authors upon reasonable request.

## Code availability

The SVR-based proteomic age prediction pipeline is available at https://github.com/marlondms/AI-ON-SKIN/ and has been archived on Zenodo^50^. The pipeline was implemented in Python 3.8 using scikit-learn, pandas, NumPy, and matplotlib.
