# Supplementary material for: Quinoa bioester application shifts human skin proteome toward molecular profiles associated with younger age
Source: Commun Biol. 2026 Apr 9;9:775. doi: 10.1038/s42003-026-10006-4 (PMC13247230; doi:10.1038/s42003-026-10006-4)
Supplement: Supplementary file 2 — Supplementary Information [file 42003_2026_10006_MOESM2_ESM.pdf]

Supplementary Note 1

Approved Study Protocol

CAAE 38352020.8.0000.5248

Parecer Consubstanciado do CEP No. 4.971.427

Research Ethics Committee of the Instituto Oswaldo Cruz,

Fundacao Oswaldo Cruz (CEP FIOCRUZ/IOC)

Approved: September 13, 2021

Contents:

Part 1: English Translation (pages 2-5)

Part 2: Original Portuguese Version (pages 6-11)

DETAILED OPINION OF THE RESEARCH ETHICS COMMITTEE

RESEARCH DATA

Opinion Number: 4.971.427 **CAAE Number:** 38352020.8.0000.5248 **Project Title:** Molecular Evaluation of the Skin **Principal Researcher:** Amanda Caroline Camillo de Andrade **Research Area:** Genomics, Proteomics, and Bioinformatics **Version:** 3 **Submitting Institution:** Fundação Oswaldo Cruz (Fiocruz) **Sponsor:** Fundação Oswaldo Cruz (Fiocruz)

SUBMISSION DATA

Submission Type: Amendment **Date of Submission:** 08/23/2021 **Document Type:** Amendment **Opinion Date:** 09/13/2021 **Status:** Approved **Meeting Number:** 295th Ordinary Meeting

---

PROJECT DETAILS

Introduction:

The skin is an essential organ for the regulation of various functions in the body, serving as an interface between the internal and external environment. With aging, skin cells are progressively damaged, affecting the normal organization of the skin and its repair capacity.

Primary Objective:

To establish characteristic protein profiles of forearm skin from research participants aged between 20 and 80 years, with phototype lower than IV, who undergo the microdermabrasion procedure.

Secondary Objectives:

- Identify characteristic proteins for each age group studied, correlating the age of research participants with skin aging;
- Elucidate skin quality based on the proteomic profile of each age;
- Understand the molecular mechanisms associated with skin aging;
- Analyze the molecular effects of Quinoa Bioester when applied to the skin;
- Investigate the efficacy of the cosmetic product on the skin;
- Correlate the effects of Quinoa Bioester with the prevention of premature skin aging.

RISK AND BENEFIT ASSESSMENT

Risks:

The molecular analysis of the samples proposed in this project will pose minimal risk to the research participant, as they will undergo a non-invasive microdermabrasion procedure aimed at the superficial removal of the stratum corneum of the epidermis, which is a routine technique widely used in the aesthetics field, with no alteration to any standard protocol.

The risks of the procedure include mild local discomfort, slight swelling, and increased sensitivity following the procedure. Should this occur, a calming cosmetic product (Eccos Calming Mask) will be applied to reduce the effects.

The home-care application of the cosmetic product containing Quinoa Bioester will pose minimal risk to the participant, as it is formulated at its commercial concentration, which has been validated by dermatologists.

The leakage of information that could compromise the anonymity of the participants could represent a potential psychological risk. To prevent such a situation, research participants will be identified by codes, and the information obtained will be encrypted with passwords.

Benefits:

The microdermabrasion procedure provides an improvement in skin appearance by stimulating collagen production, improving the permeation of cosmetic products, removing dead cells, lightening pigmentation spots, reducing acne scars, and assisting in cellular renewal. Furthermore, it is a costly procedure typically charged at approximately R$ 400.00 in aesthetic clinics or spas, and the participants will bear no cost for its performance. The benefits of this research will be indirect for the participants, as this is a basic and initial study aimed at characterizing the molecular profile of the skin associated with aging.

Here's Parts 3 and 4:

METHODOLOGY

Study Design:

This is a prospective cross-sectional study involving sixty research participants who will undergo a non-invasive microdermabrasion procedure aimed at the superficial removal of the stratum corneum of the epidermis, followed by the at-home application of a cosmetic product containing Quinoa Bioester at 0.1% on one forearm and a cosmetic

product without the active ingredient (placebo) on the other forearm, for four consecutive weeks.

Inclusion Criteria:

Female research participants with phototype lower than IV, aged between 20 and 80 years, who do not have pathologies on the arms and who agree to participate in the study by signing the Informed Consent Form.

Exclusion Criteria:

Participants with skin lesions or inflammation, age below 20 years or above 80 years, phototype greater than IV, male individuals, and participants who do not agree with the procedure and do not sign the ICF.

Primary Endpoint:

To produce greater knowledge of the molecular profile related to skin aging.

Budget:

This project will be funded by the Carlos Chagas Institute / FIOCRUZ through the Objectives and Goals Plan (POM) and the Technological Development Program in Health Inputs / PDTIS. In addition, funding will be provided by the National Council for Scientific and Technological Development / CNPq, Coordination for the Improvement of Higher Education Personnel, and Grupo O Boticário. Total budget: R$ 22,600.00.

Privacy and Confidentiality of Research Participant Data:

Research participants will be identified solely by codes. The researchers are committed to maintaining the confidentiality of the information obtained in order to guarantee the privacy of the research participants. The information obtained will be encrypted with passwords to prevent possible information leakage. No access by third parties will be permitted, ensuring full protection against any type of discrimination and/or prejudice.

COMMITTEE OPINION

The amendment submitted by the researcher was analyzed and is in accordance with the ethical requirements established by National Health Council Resolution CNS No. 466/2012.

The following modifications were approved:

1. Change of active ingredient from Vitamin C 10% to Quinoa Bioester 0.1%;
2. Change of collection region from the face to the forearm;

3. Increase in the number of research participants from 50 to 60;
4. Change of collection site to the Carlos Chagas Institute, Paraná.

The committee verified that the modifications do not alter the risk-benefit assessment of the study and that the Informed Consent Form was appropriately updated to reflect the approved changes.

Final Decision: APPROVED

Requires review by CONEP: No

Recommendations:

The researcher must submit partial reports annually and a final report within 30 to 60 days of the completion of the study, via Plataforma Brasil.

Signed by:

Celeste da Silva Freitas de Souza Coordinator, Research Ethics Committee CEP-Fiocruz/IOC

Date: September 13, 2021

PARECER CONSUBSTANCIADO DO CEP

DADOS DA EMENDA

Título da Pesquisa: AVALIAÇÃO MOLECULAR DA PELE
Pesquisador: AMANDA CAROLINE CAMILLO DE ANDRADE
Área Temática:
Versão: 3
CAAE: 38352020.8.0000.5248
Instituição Proponente: FUNDACAO OSWALDO CRUZ
Patrocinador Principal: FUNDACAO OSWALDO CRUZ

DADOS DO PARECER

Número do Parecer: 4.971.427

Apresentação do Projeto:
Este parecer esta sendo redigido para analisar a Emenda apresentada que vem solicitar a alteração do ativo Vitamina C 10% para o Bioéster de Quinoa 0,1%, o qual também se encontra comercializado. Nessa emenda também há a solicitação para a alteração da região de coleta do rosto para o antebraço, pois após algumas discussões em equipe, consideramos que a região do antebraço nos trará melhores resultados quando comparado com o rosto, isso porque um dos fatores que foram pontuados pela equipe é a participante já utilizar algum outro tipo de cosmético como protetor solar, maquiagem e isso vir a ocasionar variabilidade experimental. Nessa Emenda também há a Solicitação para o aumento do recrutamento de mais 10 participantes de pesquisa, para conseguirmos aumentar a abrangência das idades (20 a 80 anos) e alinhar a curva do envelhecimento da pele após a menopausa. E por fim, a alteração do local de coleta para o Instituto Carlos Chagas, Paraná, pela facilidade das voluntárias, que majoritariamente serão da Fiocruz-PR. O projeto visa a analise do extrato córneo, obtido por tratamento não invasivo, microdermoabrasão, que nessa emenda altera de 50 para 60 participantes da pesquisa, com fotótipo menor que IV e de diferentes faixas etárias (20 -80 anos). Este projeto se baseia na necessidade em desenvolver uma resposta de saúde pública não somente ao envelhecimento, mas também á demandas como recuperação e alterações fisiológicas da pele. Uma vez que no envelhecimento, as células da pele são progressivamente danificadas afetando a organização

normal da pele e sua capacidade de reparo. Fatores extrínsecos como radiação ultravioleta, poluição e fatores intrínsecos como diabetes ou doenças vasculares podem acelerar ainda mais esse fenômeno. Vários mecanismos estão envolvidos na degradação da pele induzida pela idade e também se relacionam com feridas crônicas ou que não cicatrizam em idosos. O projeto tem como meta precípua encontrar proteínas representativas do envelhecimento da pele para cada idade estudada, permitindo uma melhor compreensão, ao nível molecular, do efeito da idade na pele, bem como a análise molecular dos efeitos do o Bioéster de Quinoa, devido seu alto poder antioxidante. Sabendo da importância da elucidação dos diferentes perfis moleculares da pele com o avanço da idade,na prevenção do envelhecimento precoce da pele.

Objetivo da Pesquisa:

Na presente Emenda há alteração do Objetivo Principal com alteração da faixa etária,e a inclusão de seis objetivos secundarios. Assim o novo Objetivo Principal passa a ser o listado abaixo:

Objetivo Principal

Estabelecer perfis proteicos característicos de diferentes peles do antebraço das participantes de pesquisa com idades entre 20 e 80 anos, com fototipo menor que IV, submetidas ao procedimento de microdermoabrasão.

Objetivo Secundário:

- Identificar proteínas características para cada grupo de idade estudado correlacionando a idade das participantes de pesquisa com o envelhecimento da pele;
- Elucidar a qualidade da pele baseada no perfil proteico de cada idade;
- Entender os mecanismos moleculares associados com o envelhecimento da pele;
- Analisar os efeitos moleculares do Bioéster de Quinoa quando aplicados sobre a pele;
- Investigar a eficácia do produto cosmético contendo Bioéster de Quinoa na pele;
- Correlacionar os efeitos da Bioéster de Quinoa com a prevenção do envelhecimento precoce da pele.

Avaliação dos Riscos e Benefícios:

A análise molecular das amostras propostas neste projeto trará um risco mínimo para a participante da pesquisa, isto porque, serão submetidas a procedimento não invasivo de microdermoabrasão com o intuito de remoção superficial do estrato córneo da epiderme, que são técnicas de rotina amplamente utilizadas na área estética, não sendo alterado nenhum protocolo

de execução. Os riscos do procedimento são; dor leve no local, pequeno inchaço, aumento da sensibilidade após o procedimento. Caso isso ocorra, será aplicado
um produto cosmético calmante (Máscara Calmante Eccos Calming), para reduzir os efeitos após o procedimento. Por isso é importante seguir as orientações do profissional que realizou o procedimento para minimizar os riscos descritos. A aplicação home care do cosmético contendo Bioéster de Quinoa trará risco mínimo ao participante, devido ser formulado na concentração comercial, sendo validada por dermatologistas quanto à estabilidade e eficácia da concentração (CAMILLO-ANDRADE et al., 2020; STUART et al.,
2019b) O vazamento de informações que possam expor o anonimato das participantes envolvidas no estudo poderá representar um potencial risco psíquico. Para evitar tal situação, as participantes da pesquisa serão identificadas por códigos, e não por qualquer outra forma, tais como nome ou registros que possam identificar a identidade dos mesmos.
Os pesquisadores se comprometem em manter o sigilo das informações obtidas a fim de garantir a privacidade das participantes de pesquisa. As informações obtidas das participantes da pesquisa serão criptografadas por senhas para evitar um possível vazamento de informações. Não será permitido acesso à terceiros (seguidores, empregadores, superiores hierárquicos), de nenhuma informação obtida da participante da pesquisa,
garantindo proteção total contra qualquer tipo de discriminação e/ou preconceito.

Benefícios:

O procedimento de microdermoabrasão proporciona uma melhora da aparência da pele, devido estimular a produção de colágeno, melhora a permeação de produtos cosméticos, remoção das células mortas, clarear manchas de pigmentação, reduzir cicatrizes de acne e auxiliar na
renovação celular. Além disso, é um procedimento dispendioso tipicamente cobrado em torno de R$ 400,00 em clínicas de estéticas ou spas, e as participantes não terão nenhum custo para a realização do mesmo. Porém os benefícios dessa pesquisa serão indiretos para as participantes, uma vez que, trata-se de uma pesquisa básica e inicial para caracterizar o perfil molecular da pele associado ao envelhecimento. Os pesquisadores se comprometem em encaminhar os resultados da pesquisa para publicação, com os devidos créditos aos pesquisadores associados e ao pessoal técnico integrante do projeto, mantendo integramente o anonimato da participante da pesquisa. Os pesquisadores comunicarão às autoridades competentes, bem como aos órgãos legitimados pelo Controle Social, os resultados e/ou os achados da pesquisa, sempre que esses puderem

Endereço: Av. Brasil 4036, sala 705 - 7º andar (Campus Expansão)
Bairro: Manguinhos **CEP:** 21.040-361
UF: RJ **Município:** RIO DE JANEIRO
Telefone: (21)3882-9011 **Fax:** (21)2561-4815 **E-mail:** cepfiocruz@ioc.fiocruz.br

contribuir para a melhoria das condições de vida da coletividade, preservando, porém, a imagem e assegurando que as participantes da pesquisa não sejam estigmatizados.

Comentários e Considerações sobre a Pesquisa:

O projeto está sendo desenvolvido por uma Equipe que apresenta expertise para desenvolver uma pesquisa que apresenta relevância em vários âmbitos: estético, social, biológico inclusive financeiro.

Considerações sobre os Termos de apresentação obrigatória:

Para esta Emenda foram apresentados os seguintes documentos, e os mesmos estão em conformidade com a Resolução CNS466/12:

- PB_INFORMAÇÕES_BÁSICAS_1790264_E1.pdf
- TCLE_emenda_.pdf/.docx
- Justificativa_adendo.pdf
- Folhaderosto_adendo.pdf
- Projeto_18082021_adendo_.pdf
- Infra_FIO.pdf

Recomendações:

Apresentar o relatório de atividades já desenvolvidas. Ressaltamos que isso é necessário para que o sistema CEP/CONEP possa estar atualizado.

Conclusões ou Pendências e Lista de Inadequações:

A solicitação de Emenda se apresenta em conformidade e se encontra Aprovado para dar continuidade as atividades.

Considerações Finais a critério do CEP:

Diante do exposto, o Comitê de Ética em Pesquisa do Instituto Oswaldo Cruz (CEP FIOCRUZ/IOC) em sua 295a Reunião Ordinária, realizada em 13.09.2021, de acordo com as atribuições definidas na Resolução CNS 466/2012, manifesta-se pela aprovação do projeto de pesquisa proposto.

Este CEP ressalta a importância do envio dos relatórios parciais e final, sendo uma das responsabilidades assumidas pelo pesquisador ao submeter o seu projeto para apreciação (Resolução CNS 466/2012, XI.2.d e Resolução CNS 510/2016, Art. 28, V).

O Relatório Parcial deverá ser encaminhado anualmente e,

O Relatório de Conclusão (Final) deverá ser enviado aproximadamente em 30 a 60 dias após o término do projeto. Ambos os tipos de relatórios deverão ser apresentados via Plataforma Brasil,

no modo/ferramenta "Notificação".

A observância dos prazos de envio dos relatórios parciais ou finais é estritamente de responsabilidade do pesquisador. A não obediência aos prazos estipulados poderá implicar a NÃO APROVAÇÃO dos relatórios.

Este parecer foi elaborado baseado nos documentos abaixo relacionados:

Tipo Documento	Arquivo	Postagem	Autor	Situação
Informações Básicas do Projeto	PB_INFORMAÇÕES_BÁSICAS_1790264_E1.pdf	19/08/2021 20:38:07		Aceito
TCLE / Termos de Assentimento / Justificativa de Ausência	TCLE_emenda_.pdf	19/08/2021 20:37:12	AMANDA CAROLINE CAMILLO DE ANDRADE	Aceito
Outros	Justificativa_adendo.pdf	19/08/2021 14:54:45	AMANDA CAROLINE CAMILLO DE ANDRADE	Aceito
Folha de Rosto	Folhaderosto_adendo.pdf	18/08/2021 19:31:27	AMANDA CAROLINE CAMILLO DE ANDRADE	Aceito
Brochura Pesquisa	Projeto_18082021_adendo_.pdf	18/08/2021 13:45:23	AMANDA CAROLINE CAMILLO DE ANDRADE	Aceito
Declaração de Instituição e Infraestrutura	Infra_FIO.pdf	18/08/2021 13:43:43	AMANDA CAROLINE CAMILLO DE ANDRADE	Aceito
TCLE / Termos de Assentimento / Justificativa de Ausência	TCLE_emenda.docx	18/08/2021 13:43:05	AMANDA CAROLINE CAMILLO DE ANDRADE	Aceito
Outros	Responsabilidade.pdf	18/10/2020 18:03:01	AMANDA CAROLINE CAMILLO DE ANDRADE	Aceito
Outros	CartaOrientador.pdf	18/10/2020 18:02:34	AMANDA CAROLINE CAMILLO DE ANDRADE	Aceito
Outros	Carta_resposta18102020_assinada_.pdf	18/10/2020 18:02:11	AMANDA CAROLINE CAMILLO DE ANDRADE	Aceito
Outros	Carta_resposta18102020.docx	18/10/2020 18:01:52	AMANDA CAROLINE CAMILLO DE ANDRADE	Aceito

Endereço: Av. Brasil 4036, sala 705 - 7º andar (Campus Expansão)
Bairro: Manguinhos **CEP:** 21.040-361
UF: RJ **Município:** RIO DE JANEIRO
Telefone: (21)3882-9011 **Fax:** (21)2561-4815 **E-mail:** cepfiocruz@ioc.fiocruz.br

TCLE / Termos de Assentimento / Justificativa de Ausência	TCLE_18102020.docx	18/10/2020 17:56:07	AMANDA CAROLINE CAMILLO DE ANDRADE	Aceito
Projeto Detalhado / Brochura Investigador	Projeto_18102020.docx	18/10/2020 17:55:36	AMANDA CAROLINE CAMILLO DE ANDRADE	Aceito
Outros	ficha_anamnese_timbrado.pdf	21/09/2020 23:32:12	AMANDA CAROLINE CAMILLO DE ANDRADE	Aceito
Outros	Confidencialidade.pdf	21/09/2020 23:31:51	AMANDA CAROLINE CAMILLO DE ANDRADE	Aceito
Projeto Detalhado / Brochura Investigador	Projeto_21092020.pdf	21/09/2020 23:30:04	AMANDA CAROLINE CAMILLO DE ANDRADE	Aceito
TCLE / Termos de Assentimento / Justificativa de Ausência	TCLE_21092020.pdf	21/09/2020 23:29:12	AMANDA CAROLINE CAMILLO DE ANDRADE	Aceito

Situação do Parecer:
Aprovado

Necessita Apreciação da CONEP:
Não

RIO DE JANEIRO, 13 de Setembro de 2021

Assinado por:
Celeste da Silva Freitas de Souza
(Coordenador(a))

Endereço: Av. Brasil 4036, sala 705 - 7º andar (Campus Expansão)
Bairro: Manguinhos **CEP:** 21.040-361
UF: RJ **Município:** RIO DE JANEIRO
Telefone: (21)3882-9011 **Fax:** (21)2561-4815 **E-mail:** cepfiocruz@ioc.fiocruz.br
