# Supplementary material for: Quinoa bioester application shifts human skin proteome toward molecular profiles associated with younger age
Source: Commun Biol. 2026 Apr 9;9:775. doi: 10.1038/s42003-026-10006-4 (PMC13247230; doi:10.1038/s42003-026-10006-4)
Supplement: Supplementary file 3 — Description of Additional Supplementary Files [file 42003_2026_10006_MOESM3_ESM.pdf]

Description of Additional Supplementary File

File name: Supplementary data 1
Description: Source data underlying the graphs in Figures 3 and 4. Tab 1 contains chronological age and predicted proteomic age for each participant (Figure 3). Tab 2 contains predicted proteomic ages for bioactive- and vehicle-applied forearms per participant, stratified by age group (Figure 4).

File name: Supplementary data 2
Description: Quality control assessments

File name: Supplementary data 3
Description: Protein identifications (PatternLab for Proteomics V)

File name: Supplementary data 4
Description: p-values from the paired analysis between quinoa bioester and vehicle treatments

File name: Supplementary data 5
Description: Skin hydration measurement data
