# Supplementary material for: Quinoa bioester application shifts human skin proteome toward molecular profiles associated with younger age
Source: Commun Biol. 2026 Apr 9;9:775. doi: 10.1038/s42003-026-10006-4 (PMC13247230; doi:10.1038/s42003-026-10006-4)
Supplement: Supplementary file 9 — Reporting summary [file 42003_2026_10006_MOESM9_ESM.pdf]

nature portfolio

Corresponding author(s): Marlon Dias Mariano Santos (marlondms@gmail.com) e Paulo Costa Carvalho (paulo@pcarvalho.com)

Last updated by author(s): Oct 6, 2025



# Reporting Summary

Nature Portfolio wishes to improve the reproducibility of the work that we publish. This form provides structure for consistency and transparency in reporting. For further information on Nature Portfolio policies, see our Editorial Policies and the Editorial Policy Checklist.

## Statistics

For all statistical analyses, confirm that the following items are present in the figure legend, table legend, main text, or Methods section.

| n/a | Confirmed | |
|---|---|---|
| ☐ | ☒ | The exact sample size (*n*) for each experimental group/condition, given as a discrete number and unit of measurement |
| ☐ | ☒ | A statement on whether measurements were taken from distinct samples or whether the same sample was measured repeatedly |
| ☐ | ☒ | The statistical test(s) used AND whether they are one- or two-sided<br>*Only common tests should be described solely by name; describe more complex techniques in the Methods section.* |
| ☐ | ☒ | A description of all covariates tested |
| ☐ | ☒ | A description of any assumptions or corrections, such as tests of normality and adjustment for multiple comparisons |
| ☐ | ☒ | A full description of the statistical parameters including central tendency (e.g. means) or other basic estimates (e.g. regression coefficient) AND variation (e.g. standard deviation) or associated estimates of uncertainty (e.g. confidence intervals) |
| ☐ | ☒ | For null hypothesis testing, the test statistic (e.g. *F*, *t*, *r*) with confidence intervals, effect sizes, degrees of freedom and *P* value noted<br>*Give P values as exact values whenever suitable.* |
| ☒ | ☐ | For Bayesian analysis, information on the choice of priors and Markov chain Monte Carlo settings |
| ☐ | ☒ | For hierarchical and complex designs, identification of the appropriate level for tests and full reporting of outcomes |
| ☒ | ☐ | Estimates of effect sizes (e.g. Cohen's *d*, Pearson's *r*), indicating how they were calculated |

*Our web collection on statistics for biologists contains articles on many of the points above.*

## Software and code

Policy information about availability of computer code

| | |
|---|---|
| Data collection | Mass spectrometer data collection was controlled by the Xcalibur 4.1 data system (Thermo Fisher®). Skin hydration was measured using the SkinUp® digital analyzer. |
| Data analysis | Proteomic data analysis was conducted using PatternLab for proteomics V software, which is freely accessible at https://www.patternlabforproteomics.org. Quality control was assessed with RawVegetable. The machine learning model was built using Python 3.8 with the scikit-learn, pandas, and NumPy libraries. The custom code is available on GitHub at https://github.com/marlondms/AI-ON-SKIN/. |

For manuscripts utilizing custom algorithms or software that are central to the research but not yet described in published literature, software must be made available to editors and reviewers. We strongly encourage code deposition in a community repository (e.g. GitHub). See the Nature Portfolio guidelines for submitting code & software for further information.

## Data

Policy information about availability of data

All manuscripts must include a data availability statement. This statement should provide the following information, where applicable:

- Accession codes, unique identifiers, or web links for publicly available datasets
- A description of any restrictions on data availability
- For clinical datasets or third party data, please ensure that the statement adheres to our policy

The mass spectrometry proteomics data have been deposited to the ProteomeXchange Consortium via the PRIDE partner repository with the dataset identifier PXD062216.

## Research involving human participants, their data, or biological material

Policy information about studies with human participants or human data. See also policy information about sex, gender (identity/presentation), and sexual orientation and race, ethnicity and racism.

| | |
|---|---|
| Reporting on sex and gender | The study exclusively included female participants. Therefore, the findings and conclusions of this research apply only to females. Biological sex was used as an inclusion criterion for the study. |
| Reporting on race, ethnicity, or other socially relevant groupings | Participants were categorized by Fitzpatrick skin phototypes, with only individuals having types II to IV being included. This classification was relevant to control for variability in skin aging, which can differ significantly across phototypes and ethnic backgrounds. The study acknowledges that its findings require validation in more diverse populations. |
| Population characteristics | The study included 60 female participants, with one participant for each chronological age from 20 to 80 years old. All participants had Fitzpatrick skin phototypes between II and IV. Exclusion criteria included skin diseases, smoking, diabetes, and pregnancy. |
| Recruitment | Participants were openly recruited volunteers who were screened using a standardized form and ID verification. The study employed a "single-year quota sampling" strategy to enroll exactly one participant per age from 20 to 80, ensuring a uniform age distribution. |
| Ethics oversight | The study protocol was approved by the ethics committee under the reference number CAAE 38352020.8.0000.5248. |

Note that full information on the approval of the study protocol must also be provided in the manuscript.

# Field-specific reporting

Please select the one below that is the best fit for your research. If you are not sure, read the appropriate sections before making your selection.

☒ Life sciences ☐ Behavioural & social sciences ☐ Ecological, evolutionary & environmental sciences

For a reference copy of the document with all sections, see nature.com/documents/nr-reporting-summary-flat.pdf

# Life sciences study design

All studies must disclose on these points even when the disclosure is negative.

| | |
|---|---|
| Sample size | The sample size of 60 participants was not determined by a statistical power calculation but by a prospective "single-year quota sampling" strategy. The objective was to enroll one unique participant for each chronological age from 20 to 80 years. This design was chosen to create a broad and evenly distributed age cohort, which is essential for training a machine learning model to predict age from proteomic data |
| Data exclusions | No participants or samples were excluded from the study analysis. However, during the proteomic data processing stage, specific protein identifications were excluded based on pre-established quality control criteria. Identifications with a confidence score below 2 or a mass deviation of more than 10 ppm from the theoretical mass were removed from the final dataset to ensure high data quality. |
| Replication | o ensure analytical reproducibility, all skin samples were analyzed by mass spectrometry in technical duplicates. Furthermore, the study's paired design, where each participant's placebo-treated forearm served as a direct control for their bioactive-treated forearm, is a robust method for verifying findings by minimizing inter-individual variability. The authors note that while technical replication was performed, the study did not include biological replicates for each age (i.e., multiple participants of the same age). |
| Randomization | The study employed randomization for treatment allocation. The assignment of which forearm (left or right) received the active quinoa bioester formulation versus the placebo was randomized among the participants. This was done to control for potential biases introduced by physiological asymmetries or side dominance. |
| Blinding | The study was single-blinded. The participants were blinded to the treatment, as the active and placebo formulations were identical in appearance and texture. |

# Reporting for specific materials, systems and methods

We require information from authors about some types of materials, experimental systems and methods used in many studies. Here, indicate whether each material, system or method listed is relevant to your study. If you are not sure if a list item applies to your research, read the appropriate section before selecting a response.

## Materials & experimental systems

| n/a | Involved in the study |
|---|---|
| ☒ | ☐ Antibodies |
| ☒ | ☐ Eukaryotic cell lines |
| ☒ | ☐ Palaeontology and archaeology |
| ☒ | ☐ Animals and other organisms |
| ☐ | ☒ Clinical data |
| ☒ | ☐ Dual use research of concern |
| ☒ | ☐ Plants |

## Methods

| n/a | Involved in the study |
|---|---|
| ☒ | ☐ ChIP-seq |
| ☒ | ☐ Flow cytometry |
| ☒ | ☐ MRI-based neuroimaging |

# Clinical data

Policy information about clinical studies

All manuscripts should comply with the ICMJE guidelines for publication of clinical research and a completed CONSORT checklist must be included with all submissions.

| | |
|---|---|
| Clinical trial registration | N/A |
| Study protocol | The complete methodology, including participant criteria, sample collection, proteomic analysis, and statistical approach, is detailed in the "Material and Methods" section of the paper . |
| Data collection | Data collection occurred in a single city. Skin samples were collected from the inner forearm of each participant at two time points: once at baseline before the study began, and a second time after a 30-day treatment period. The specific recruitment and data collection dates are not specified in the manuscript . |
| Outcomes | The primary outcome was the change in the skin's proteomic profile following treatment. This was assessed by quantifying changes in protein abundance using mass spectrometry and by calculating a "predicted proteomic age" with a machine learning model (SVR) trained on the baseline proteomic data. The secondary outcome was skin hydration, which was measured using a digital bioelectrical impedance analyzer before and after the treatment period. |

# Plants

| | |
|---|---|
| Seed stocks | N/A |
| Novel plant genotypes | N/A |
| Authentication | N/A |
