# Supplementary material for: Quinoa bioester application shifts human skin proteome toward molecular profiles associated with younger age
Source: Commun Biol. 2026 Apr 9;9:775. doi: 10.1038/s42003-026-10006-4 (PMC13247230; doi:10.1038/s42003-026-10006-4)
Supplement: Supplementary file 1 — Transparent Peer Review file [file 42003_2026_10006_MOESM1_ESM.pdf]

nature portfolio

Peer Review File

Quinoa bioester application shifts human skin proteome toward molecular profiles associated with younger age

Corresponding Author: Dr Paulo Carvalho

This file contains all reviewer reports in order by version, followed by all author rebuttals in order by version.

Version 0:

Reviewer comments:

Reviewer #1

(Remarks to the Author)
The manuscript “Quinoa Bioester Treatment Shifts Human Skin Proteome Toward Younger Molecular Signatures” by Camillo-Andrade et al. describes a machine learning approach using Support Vector Regression to predict biological skin age from proteomic profiles, enabling objective assessment of anti-aging interventions. The integration of proteomics with machine learning represents a novel and unbiased method for evaluating treatment effectiveness, and the findings suggest that quinoa bioester treatment can shift molecular signatures toward a younger state.

Several experiments, however, need further clarification. Aging is a complex process that involves changes not only at the proteomic level, but also at the genomic, epigenetic, and other molecular levels. In the context of skin aging, morphological changes should also be considered alongside molecular alterations. Does this correlate with any visible or clinical improvement in skin condition? Could the authors also characterize the treatment effectiveness by clinical scales for measuring skin aging?

Additionally, while the treatment duration was 30 days—are there data on how long the molecular age shift persists after stopping treatment?

Reviewer #2

(Remarks to the Author)

This study developed a proteomics aging clock for human skin and concluded that quinoa bioester treatment shifts the human skin proteome toward younger states. It is a very interesting study, however, we have some concerns, and questions, detailed, below.

1. Fig. 4 shows that the median predicted proteomic age of the post-treatment samples much higher than the median chronological age of the placebo groups and the young bioactive group. Does it make sense that the placebo aged the skin? We rather think that the proteomic aging clock highly overfit as it performed much worse in the unseen samples. Maybe a more straightforward and usual method would be better (e.g., glmnet as it contain a built-in parameter optimization; it selects the important features too).

2. It may be problematic to train and test the models on the same individuals (same forearm pre-treatment and post-treatment). Usually, samples of a given subject are not recommended for use in both the training and testing sets.

3. It seems that the clock predicted very large skin age (140 years in some cases) for the placebo (and even some bioactive) samples, but not for the pre-treatment samples. Have the authors checked the possible reasons/mechanisms behind this phenomenon?

4. It would be useful to see performance metrics for the post-treatment samples too (placebo and bioactive samples separately).

5. It would be also useful to explore the most important features in the model to see which mechanisms of aging the clock captures. How much do these proteins overlap with the differentially expressed proteins?

6. It would be useful to illustrate in a figure the abundance change of proteins revealed by the paired proteomic analysis to support the findings.

7. It would be interesting to see how many proteins remained after feature elimination. A large number of proteins are necessary to explain the aging process or a few is enough to capture it?

8. Showing the predictions paired in Fig. 4.(by using paired box plots between the treated and placebo samples would provide additional, useful information. Also, what is the number of samples in each groups?

9. How was the hyperparameter optimization done? A clear description is needed. Which samples (how many) was used for the optimization, which parameters where used, which was the best? The same is true for the feature selection procedure.

10. Was the min-max scaling done separately on the training and post-treatment data to avoid information leakage?

11. in line 362: How did the authors find exactly one patient for each year of age?

12. in line 373: How did the authors make sure that participants did not switch the fomula? Were the containers labeled such as “for left arm” or “for right arm”?

13. in line 470: What is "relative" biological age? Why relative?

14. Did you see the difference of the rejuvenation by eyes (visually)? Some pictures would be useful.

15. Will the datasets be available? It would be very useful. The PRIDE ID is not available yet. "Sorry, no projects found for search term PXD062216 and the current set of active filters."

Reviewer #3

(Remarks to the Author)
I co-reviewed this manuscript with one of the reviewers who provided the listed reports. This is part of the Communications Biology initiative to facilitate training in peer review and to provide appropriate recognition for Early Career Researchers who co-review manuscripts.

Reviewer #4

(Remarks to the Author)
Dear authors,
This study introduces a machine learning approach using Support Vector Regression (SVR) to predict biological skin age based on proteomic profiles. In a randomized, placebo-controlled trial involving 61 women aged 20–80, a 30-day topical treatment with quinoa bioester upregulated proteins associated with skin barrier function, antioxidant defense, and protease inhibition. The SVR model revealed that quinoa-treated skin appeared 11–16 years younger than placebo-treated skin in participants aged 50 and above. These findings provide a quantitative framework for evaluating anti-aging therapies and highlight quinoa bioester as a promising rejuvenating agent. However, I believe that some revisions and suggestions could further enhance the clarity, readability, and overall impact of the manuscript. These adjustments would help make the paper more accessible to a broader audience and better aligned with the standards of Communications Biology.
1. Please ensure consistency in terminology throughout the manuscript especially in the use of terms like SVR.
2. I recommend briefly discussing the biological relevance of the quinoa bioester used in the SVR prediction, particularly in relation to its potential in vivo applications.
3. Could you please clarify the source of the drawing and photograph in Figure 1? If the image was created by you or one of your co-authors, kindly cite the software used in the figure legend.
4. Figure 3 description effectively explains the scatter plot and the use of the Leave-Age-Out (LAO) validation approach, which is appropriate for assessing age prediction performance. However, I recommend briefly clarifying why the LAO method was chosen over other cross-validation techniques, as this would help justify its use. Additionally, specifying the sample size (number of individuals) and providing confidence intervals for the MAE and R^2 would improve the robustness and interpretability of the model performance metrics. Lastly, consider stating whether any age-related bias or deviation from the y = x line was observed across the age spectrum.
5. In Figure 4, the left panel represents participants aged under 50, while the right panel represents participants aged 50 and older. Boxplots compare the predicted ages for bioactive-treated and placebo-treated arms," is generally clear but could be improved by specifying what the predicted age refers to (i.e., biological skin age) and whether the differences between groups are statistically significant. Including sample sizes and p-values in the figure legend or accompanying text would enhance interpretability and rigor.
6. Consider discussing whether the observed proteomic changes are expected to be transient or long-lasting, and whether continued application might enhance or diminish these effects over time. This is important for assessing the sustainability of the treatment’s impact.
7. Were the proteomic responses consistent across different age groups (e.g., under 50 vs. over 50)? Since aging is a major

factor in skin biology, discussing whether certain protein changes were more prominent in older participants would add valuable depth to the interpretation.
8. The discussion could be enhanced by synthesizing the observed changes into a unified mechanistic hypothesis or pathway-level interpretation for example, how quinoa bioester might coordinately regulate epidermal differentiation, oxidative defense, and barrier restoration.
9. The reduction in predicted proteomic age (11–16 years) is significant and compelling. However, the manuscript could be strengthened by discussing how such changes compare to known clinical or phenotypic measures of rejuvenation. For instance, are there benchmarks or prior studies with similar anti-aging effects (even if non-proteomic) that can serve as context?
10. Although the study was conducted in women, and menopause is mentioned, it would be valuable to discuss whether hormonal status was assessed or controlled for, and whether the findings may translate to males or postmenopausal populations beyond age grouping. How might these findings apply to individuals with different skin types, and ethnic backgrounds?
11. The manuscript notably lacks a conclusion section, which is essential for summarizing the key findings, reinforcing the scientific contributions, and outlining the broader implications of the work.
12. A few additional experiments or analyses could further strengthen the scientific rigor and impact of the study, particularly given the standards of Communications Biology.
(i) Exploring different concentrations of quinoa bioester or sampling at multiple time points during treatment could reveal the dynamics and optimal conditions of its effect on skin proteomics.
(ii) Conducting a systems-level analysis (e.g., gene ontology or pathway enrichment analysis) of the differentially abundant proteins would provide mechanistic insights and strengthen the biological interpretation of the findings.
13. The manuscript does not exhibit any major technical or conceptual flaws that would prohibit its publication. The study is methodologically sound and presents an innovative approach using support vector regression (SVR) on proteomic data to estimate biological skin age and evaluate the rejuvenating potential of quinoa bioester.

Version 1:

Reviewer comments:

Reviewer #1

(Remarks to the Author)
I have reviewed the revised manuscript and am satisfied with the authors’ responses. I have no further comments.

Reviewer #2

(Remarks to the Author)
The concerns raised by us previously, have mostly been addressed. The goal of the study and hence the chosen methodology is now more understandable. However, we feel that stating the 11-16 years reduction in proteomic age is not established. The authors point to the domain shift causing the post-treatment prediction shifting upwards compared to the untreated predictions, and because of this the absolute age estimations are not reliable. The authors state that "our primary contribution is demonstrating differential treatment effects rather than precise absolute age estimation in treated skin", which we highly support. In order to align with this, we suggest refining the conclusions on exact age reduction.

Reviewer #3

(Remarks to the Author)
I co-reviewed this manuscript with one of the reviewers who provided the listed reports. This is part of the Communications Biology initiative to facilitate training in peer review and to provide appropriate recognition for Early Career Researchers who co-review manuscripts.

Reviewer #4

(Remarks to the Author)

Open Access This Peer Review File is licensed under a Creative Commons Attribution 4.0 International License, which permits use, sharing, adaptation, distribution and reproduction in any medium or format, as long as you give appropriate credit to the original author(s) and the source, provide a link to the Creative Commons license, and indicate if changes were made.
In cases where reviewers are anonymous, credit should be given to 'Anonymous Referee' and the source.
The images or other third party material in this Peer Review File are included in the article's Creative Commons license, unless indicated otherwise in a credit line to the material. If material is not included in the article's Creative Commons license and your intended use is not permitted by statutory regulation or exceeds the permitted use, you will need to obtain permission directly from the copyright holder.
To view a copy of this license, visit https://creativecommons.org/licenses/by/4.0/

Dear Dr., Bircan,

We thank you and the reviewers for reviewing our manuscript and appreciate the constructive feedback and the opportunity to strengthen our work through revision. We are encouraged that the reviewers found our integration of proteomics with machine learning to be novel and of interest; in particular, Reviewer #4 noted no major technical or conceptual flaws that would prohibit publication. We have carefully considered all comments and have substantially revised the manuscript to address the constructive suggestions. Please find below each point raised by the reviewers together with the actions taken and justifications.

Reviewer #1 (Remarks to the Author):

The manuscript "Quinoa Bioester Treatment Shifts Human Skin Proteome Toward Younger Molecular Signatures" by Camillo-Andrade et al. describes a machine learning approach using Support Vector Regression to predict biological skin age from proteomic profiles, enabling objective assessment of anti-aging interventions. The integration of proteomics with machine learning represents a novel and unbiased method for evaluating treatment effectiveness, and the findings suggest that quinoa bioester treatment can shift molecular signatures toward a younger state.

We sincerely thank the reviewer for recognizing the novelty of our unbiased approach and for the insightful understanding that this provides a framework for evaluating topical treatments.

Several experiments, however, need further clarification. Aging is a complex process that involves changes not only at the proteomic level, but also at the genomic, epigenetic, and other molecular levels. In the context of skin aging, morphological changes should also be considered alongside molecular alterations.

Does this correlate with any visible or clinical improvement in skin condition?

We agree that aging encompasses multiple molecular levels beyond proteomics. We did observe visible improvements that correlated with our proteomic findings. Besides, participants independently reported noticeable improvements in skin texture and hydration, albeit claiming in both arms, which, as noted in our manuscript, the placebo also contained hydrating base ingredients.

Could the authors also characterize the treatment effectiveness by clinical scales for measuring skin aging?

We thank the reviewer for this suggestion. Although classical clinical aging scales were not part of our design, we conducted quantitative skin hydration assessments using the SkinUp®, a digital analyzer based on bioelectrical impedance. Increased hydration reduces skin impedance by enabling more continuous conductive pathways formed by

water and ions. The device provides relative hydration values in arbitrary units and has been validated in vivo against the gold-standard Corneometer® CM825 (DOI: 10.1111/srt.12833). Higher values indicate greater hydration.

Briefly, skin hydration measurements in all 61 participants showed that quinoa bioester treatment produced greater improvements compared to placebo (mean Δ = 1.14 for treated vs 0.54 for placebo, *p*-value = 0.17), representing a 111% greater hydration improvement, directionally consistent with proteomic findings effect size. This data is not in the original manuscript.

It is important to note that our p-value of 0.17 exceeds the conventional 0.05 threshold; nonetheless, after reflecting in the light of the reviewer's comments, we believe these data indeed merit inclusion in our manuscript. Although not statistically significant, a p-value of 0.17 suggests a trend that would occur by chance alone in less than, roughly, one in five times, and when combined with a substantial effect size (111% greater improvement: mean Δ = 1.14 vs 0.54 arbitrary units), provides suggestive evidence worthy of report. The consistent directional improvement across participants, coupled with our statistically significant proteomic findings, provides multi-level support for the treatment's beneficial effects. We have included these hydration measurements as Supplementary Table 1 and integrated discussion of these findings into the manuscript.

Manuscript modifications made:

1. Methods section addition (Sample Collection subsection): Added after the treatment protocol description*:* "Skin hydration was quantitatively assessed using the SkinUp® digital analyzer, which measures bioelectrical impedance. This device has been validated against the gold-standard Corneometer® CM825 (DOI: 10.1111/srt.12833). Measurements were taken from the same standardized forearm areas before and after the 30-day treatment period."

2. Results section addition (new paragraph after the paired proteomic analysis): Added: "To complement our proteomic findings, we assessed skin hydration changes using bioelectrical impedance measurements. Hydration analysis across all 61 participants revealed that both the placebo and the quinoa bioester treatment produced improvements in skin hydration (mean Δ = 0.54 arbitrary units for placebo vs 1.14 for bioactive, $p = 0.17$), with the quinoa bioester group showing improvement compared to placebo. While this difference did not reach conventional statistical significance ($p > 0.05$), the consistent directional improvement suggests a meaningful biological effect. The high inter-individual variability expected given our diverse age range (20-80 years), combined with the hydrating effects of the placebo base formulation itself, makes detecting additional bioactive-specific effects particularly challenging through conventional metrics alone. Skin hydration measurement data are available in Supplementary File 4."

3. Discussion section modification: Added to the paragraph discussing treatment effects: "The proteomic age reduction observed with quinoa bioester treatment was supported by quantitative hydration measurements, which showed a 111% greater

improvement compared to placebo. This multi-level evidence—combining molecular signatures with functional skin parameters corroborates to our conclusion that quinoa bioester provides measurable skin health benefits."

Additionally, while the treatment duration was 30 days—are there data on how long the molecular age shift persists after stopping treatment?

We appreciate this important suggestion; however, our study did not include post-treatment follow-up. Such an extension would require additional skin sampling from the same participants at multiple timepoints, which presents practical challenges with volunteer retention and increases participant burden through repeated skin collection procedures. Nonetheless, we fully agree that investigating the persistence of these proteomic changes represents an important direction for future research.

Reviewer #2 (Remarks to the Author):

This study developed a proteomics aging clock for human skin and concluded that quinoa bioester treatment shifts the human skin proteome toward younger states. It is a very interesting study, however, we have some concerns, and questions, detailed, below.

We thank the reviewer for their detailed technical assessment and thoughtful questions. These important points have helped us clarify our methodology and strengthen the manuscript. We address each concern below:

1. Fig. 4 shows that the median predicted proteomic age of the post-treatment samples much higher than the median chronological age of the placebo groups and the young bioactive group. Does it make sense that the placebo aged the skin?

We appreciate the reviewer's observation. The systematic elevation in predicted ages following any topical treatment, including placebo, reflects a domain shift phenomenon in machine learning, where models encounter distributions outside their training space. Our model, trained exclusively on untreated skin, detects the proteomic alterations induced by 30 days of twice-daily application and interprets these through its learned framework. Importantly, while absolute predictions shift upward, the preserved relative difference between treatments demonstrates the model's discriminative capacity remains intact; this is a key finding. Our choice of a single linear SVR, while unable to capture all age-related non-linearities partially visible in Figure 3, was deliberate to avoid overfitting given our sample size constraints. We fully agree with the reviewer that these elevated predictions warrant discussion; nonetheless, we interpret them as the model successfully detecting that treated skin differs from untreated skin while maintaining the ability to discriminate treatment efficacy on completely new samples. As now better reflected in the revised manuscript, given several points raised by this reviewer that should be made more evident in our work, our primary contribution is demonstrating differential treatment effects rather than precise absolute age estimation in treated skin.

We rather think that the proteomic aging clock highly overfit as it performed much worse in the unseen samples. Maybe a more straightforward and usual method would be better (e.g., glmnet as it contain a built-in parameter optimization; it selects the important features too).

The model maintains predictive relationships in post-treatment data; the correlation between predicted ages for bioactive versus placebo remains consistent, with bioactive samples systematically predicted several years younger. As such, we presume that what the reviewer mentioned as poor performance is the systematic offset discussed above, not degraded predictive capability. This distinction is crucial: overfitting produces erratic, unreliable predictions on new data, whereas our model shows consistent, interpretable shifts that preserve treatment discrimination in never seen data.

The elevated ages reflect a well-documented phenomenon in machine learning, domain shift, where models encounter data from a different distribution than their training set. Our model was trained only on untreated skin. Importantly, the model's ability to discriminate between treatments remains robust, which is our primary objective.

Regarding methodology, we actually started this project with Lasso regression / elastic net, exactly as suggest; we were motivated by glmnet's elegant built-in cross-validation and feature selection. We spent considerable time with these approaches (as they were our initial preference). However, when we compared performance head-to-head, linear SVR-RFE, we gained an edged, albeit marginally. In our specific context with high-dimensional proteomic data, SVR's margin-based optimization seemed to handle the noise slightly better.

That said, the reviewer is absolutely right that both are linear methods with different flavors of regularization; yet, switching between them did not resolve the domain shift we're seeing. We chose to present just the SVR results to keep the narrative clean, but we completely understand why the glmnet is an interesting option.

2. It may be problematic to train and test the models on the same individuals (same forearm pre-treatment and post-treatment). Usually, samples of a given subject are not recommended for use in both the training and testing sets.

We appreciate the reviewer raising this important methodological consideration. We acknowledge the point of using samples from the same individuals in both training and testing phases, where independent datasets are preferred. However, we respectfully argue that our approach is both intentional and appropriate for our specific research question, which fundamentally differs from developing a deployable age prediction model.

Our group's experience with proteomic studies in human subjects has consistently demonstrated that inter-individual variability far exceeds intra-individual changes, even after interventional treatments (Brunoro et al., BMC Cancer 2019; Camillo-Andrade et al., Sci Data 2024); these manuscripts are referenced in the main text. Unlike controlled cell

culture experiments, human skin proteomics exhibits enormous biological variability driven by genetics, lifestyle, environmental exposures, and unmeasured factors. This variability is further amplified after a 30-day treatment period, where individual responses to topical formulations vary substantially based on skin barrier function, absorption rates, and metabolic differences.

Critically, our research question is not "Can we build a generalizable age predictor for any new individual?", in which the reviewer would be completely right to be concerned; but rather "Can quinoa bioester treatment induce detectable rejuvenation-associated proteomic shifts within the same individuals?" This distinction is critical and fundamental. We are using the SVR model as an analytical tool to detect within-subject treatment effects, not as a predictive model for deployment on independent populations. The paired design, where each individual serves as their own control, is essential for this purpose, as it allows us to:

1. Control for the massive inter-individual proteomic variation that would otherwise overwhelm treatment signals
2. Detect subtle but consistent treatment-induced changes that manifest differently across individuals due to baseline heterogeneity
3. Ensure that observed differences reflect treatment effects rather than population stratification or batch effects

If we had used completely independent individuals for training and testing, we would be answering a different question entirely: whether age patterns learned from one population can predict ages in another population after treatment. This would conflate two sources of variation (inter-individual differences and treatment effects) making it way harder to isolate the treatment's impact and requiring much larger cohorts which would exceed our budget by far.

We note that the 30-day temporal gap and the domain shift observed (elevated predicted ages in all treated samples) actually demonstrate that our model is not simply memorizing individual patterns. If it were, we would expect accurate age predictions post-treatment. Instead, the systematic offset indicates the model correctly identifies that treated skin differs proteomically from untreated skin, while still maintaining the ability to discriminate between treatment conditions.

We thank the reviewer for raising this important methodological point, which is a legit and fundamental consideration. This critical observation should be made evident in the main text, we now added a paragraph to the Discussion.

Manuscript modification for Discussion section: "An important consideration in our study design is the use of samples from the same individuals for both model training (pre-treatment) and testing (post-treatment). Such experimental design is motivated for detecting within-subject treatment effects rather than building a generalizable age predictor for new populations. The massive inter-individual variability in human skin proteomics, which far exceeds treatment-induced changes, necessitates paired analysis to better focus on treatment effects. We emphasize that our SVR model serves as an analytical tool for quantifying relative proteomic shifts within individuals, and not as a

deployable age prediction system. Future studies seeking to develop generalizable proteomic age clocks would require independent and unpaired validation cohorts.

3. It seems that the clock predicted very large skin age (140 years in some cases) for the placebo (and even some bioactive) samples, but not for the pre-treatment samples. Have the authors checked the possible reasons/mechanisms behind this phenomenon?

We attribute the elevated predictions to domain shift, where our model trained on untreated skin must extrapolate when encountering treated skin. Bioactive-treated samples consistently show younger predicted ages than placebo-treated samples. We could indeed use more sophisticated regressors, such as, say, a fitting a sigmoid with asymptotic behavior at the training dataset min and max, but decided to stay with the linear regression and all of its limitations in exchange for diminishing overfitting risks.

4. It would be useful to see performance metrics for the post-treatment samples too (placebo and bioactive samples separately).

We intentionally present box plots rather than traditional performance metrics (MAE, R^2) as these metrics assume accurate absolute age prediction, which is not our objective. The systematic domain shift affects both placebo and bioactive treatments equally—they were applied identically for 30 days, creating the same methodological conditions. What matters is that despite this uniform shift, bioactive samples are consistently predicted 11-16 years younger than their paired placebo controls ($p<0.01$ for ≥50 group). This differential is observed across 61 individuals, shows age-dependent magnitude (stronger in older participants where aging effects are more pronounced), and aligns with our mechanistic findings of enhanced barrier proteins and reduced oxidative stress markers. The reproducibility of this relative difference under identical domain shift conditions demonstrates genuine biological signal rather than random variation.

Here we authors joined the reviewer's question's 5 and 7 below as we believe our response addresses both questions, jointly.

5. It would be also useful to explore the most important features in the model to see which mechanisms of aging the clock captures. How much do these proteins overlap with the differentially expressed proteins? 7. It would be interesting to see how many proteins remained after feature elimination. A large number of proteins are necessary to explain the aging process or a few is enough to capture it?

This is an interesting point as we were also curious if there were a minimal set of which we could predict aging. It turns out that we were able to converge to an RFE-SVR panel of 200 without losing MAE performance, that remains stable in MAE until 120 proteins. Five proteins overlap with our paired comparison panel that follows treatment. This limited overlap is somewhat expected and the proteins are biologically meaningful in regards to aging. The SVR panel identifies proteins whose baseline levels correlate with chronological age (trained on untreated samples), while paired analysis identifies acute

treatment responses and is derived from proteomes pos-treatment. We noted that such overlapping proteins are established hallmarks of aging**:**

- **Thioredoxin**: Master antioxidant that declines with age; its upregulation suggests enhanced oxidative stress defense, a key mechanism of rejuvenation (DOI: 10.1016/j.ejphar.2022.174756 / DOI: 10.3390/antiox12051078.)
- **Kallikrein-7**: Serine protease controlling desquamation; dysregulated in aged skin causing impaired barrier function (DOI: 10.1097/JD9.0000000000000036)
- **Vinculin**: Mechanotransduction protein linking cellular adhesion to cytoskeleton; altered expression affects tissue integrity and wound healing in aging (DOI: 10.1007/s00018-017-2511-3/ DOI: 10.1083/jcb.122.6.1285).
- **Myosin**: Non-muscle myosin essential for cellular mechanics; changes affect fibroblast contractility and ECM remodeling (DOI: 10.1016/j.yexcr.2014.04.013).
- **Ganglioside GM2 activator** is associated with aging because its altered expression in aged skin has been linked to lysosomal dysfunction and impaired lipid metabolism, processes that contribute to age-related skin barrier decline (DOI: 10.1016/j.nbd.2025.106798)

6. It would be useful to illustrate in a figure the abundance change of proteins revealed by the paired proteomic analysis to support the findings.

We now highlighted that the fold changes are also available in Supplementary Table 3 in the manuscript's main text and thank the reviewer for pointing this out. As for the Figure, we prefer to maintain focus on our primary contribution, the unbiased SVR-based prediction. As such, we modified the manuscript as follows:

Manuscript alterations

Original text (end of "Paired Proteomic Analysis Reveals..." section): "A complete list of differentially abundant proteins is available in Supplementary File 3."

Modified text: "A complete list of differentially abundant proteins, including Log_2 fold changes and *p*-values identified in the paired analysis between quinoa bioester and placebo treatments, is available in Supplementary File 3. "

8. Showing the predictions paired in Fig. 4.(by using paired box plots between the treated and placebo samples would provide additional, useful information. Also, what is the number of samples in each groups?

We thank the reviewer for this suggestion. While we considered paired plots to show individual responses, we prefer to maintain separate box plots as they are simpler to interpret, emphasize population-level effects, and avoid visual clutter with 30-31 connecting lines per panel. However, we have revised the Figure 4 legend to clearly specify the sample sizes and paired nature of the data: n=30 individuals aged 20-49 years and n=31 individuals aged 50-80 years, with each participant contributing both bioactive and placebo measurements. We emphasize that although the visualization uses separate box plots, our statistical analysis appropriately employed the Wilcoxon signed-rank test for paired comparisons, accounting for the within-subject design where each participant served as their own control.

Original Fig 4 Legend: Predicted Proteomic Age of Bioactive- and Placebo-Treated Skin, Stratified by Participant Age Group. The left panel represents participants aged under 50, while the right panel represents participants aged 50 and older. Boxplots compare the predicted ages for bioactive-treated and placebo-treated arms.

Revised Figure 4 Legend: "Fig 4: Predicted Proteomic Age of Bioactive- and Placebo-Treated Skin, Stratified by Participant Age Group. The left panel represents participants aged under 50 (n=30), while the right panel represents participants aged 50 and older (n=31). Boxplots compare the predicted proteomic ages (derived from the SVR model trained on pre-treatment proteomic profiles) for bioactive-treated versus placebo-treated forearms from the same individuals after 30 days of treatment. Statistical significance was assessed using the Wilcoxon signed-rank test for paired comparisons: $p=0.14$ for the <50 group (median reduction of 11 years) and $p<0.01$ for the ≥50 group (median reduction of 16 years), comparing bioactive to placebo within each age stratum. The differences between age groups reflect the expected age-related changes in skin proteome, with older participants showing higher predicted proteomic ages in both treatment conditions*”.*

9. How was the hyperparameter optimization done? A clear description is needed. Which samples (how many) was used for the optimization, which parameters where used, which was the best? The same is true for the feature selection procedure.

One advantage of linear SVR is that it contains only the regularization parameter C, thus requiring minimal hyperparameter tuning. We tested C values of [10, 100, 1000, 10000, 100000] using Leave-Age-Out cross-validation and selected C=100000 based on minimum MAE. Details have been added to the Methods section.

Manuscript modification (Methods - SVR subsection):

Revised text: "We performed Leave-Age-Out cross-validation on pre-treatment samples (n=122) to simultaneously optimize hyperparameters and evaluate model performance. Testing regularization parameter C values of [10, 100, 1000, 10000, 100000], we selected C=100000 which achieved MAE = 7.17 years ($R^2 = 0.74$).

10. Was the min-max scaling done separately on the training and post-treatment data to avoid information leakage?

The scaler was fitted only on the training data, and these same scaling parameters were then applied to transform the post-treatment samples.

To make this more evident, we revised the manuscript as follows:

Current text (in Methods - RFE section): "Protein quantitation values were normalized to a 0-1 range using MinMax scaling to ensure all features contributed equally to the model regardless of their original scale."

Revised text with addition: "Protein quantitation values were normalized to a 0-1 range using MinMax scaling to ensure all features contributed equally to the model regardless of their original scale. The scaler was fitted exclusively on the pre-treatment training samples, and these same scaling parameters were subsequently applied to transform the post-treatment test samples, ensuring consistent normalization without incorporating any information from the test set distribution. All feature selection (RFE) were performed within a nested LAO framework; in each fold, feature selection and hyperparameter optimization were fit on the training ages only, then evaluated on the held-out age."

11. In line 362: How did the authors find exactly one patient for each year of age?

Thank you for this question. This was indeed a challenging requirement, which we anticipated and addressed through the study design. We used age-stratified quota sampling with single-year strata across the targeted age range. Recruitment was conducted via public calls for volunteers, after which respondents completed a standardized screening form. A key inclusion rule was uniqueness of year of birth: we verified year of birth using official identification and enrolled only one individual per birth-year stratum. Enrollment proceeded iteratively with continuous monitoring of the accrual log; once a stratum was filled, subsequent candidates from the same birth year were kept as alternates. If an enrolled participant withdrew before baseline assessment, the next eligible alternate from the same stratum was contacted, thereby preserving the one-per-year structure. Age was defined as completed years at the baseline (index) visit to avoid misclassification from rounding. This preplanned, criterion-based procedure ensured exactly one participant for each year of age in the final sample.

Manuscript modifications made:

1. **Methods section addition (Sample Collection subsection):**

Age-stratified enrollment. We prospectively used single-year quota sampling across the prespecified 20–80 range. Volunteers were openly recruited and screened with a standardized form, ID verification, and considering year of birth; we enrolled one

participant per birth-year. Age was defined as completed years at baseline, ensuring one participant of each age.

12. in line 373: How did the authors make sure that participants did not switch the formula? Were the containers labeled such as “for left arm” or “for right arm”?

As the reviewer correctly presumed, the containers were clearly labeled "right forearm" or "left forearm" with accompanying written instructions. Additionally, we strategically chose the volar forearm site as, among other advantages such as low sun exposure, it inherently minimizes cross-contamination risk as participants necessarily need to use different hands to apply each product.

Manuscript modification: *Added to Methods section after randomization statement:* "Formulation tubes were labeled 'right forearm' or 'left forearm' with accompanying written instructions to ensure correct application throughout the treatment period."

13. in line 470: What is "relative" biological age? Why relative?

We thank the reviewer for identifying this confusing terminology. We have revised the manuscript to consistently use "predicted biological age" throughout, which more accurately describes what our SVR model outputs—a prediction of biological age based on proteomic profiles. Even though we now make more evident that our model is not an age predictor in the manuscript but used to compare the effect of the treatments. The term "relative" was initially used to emphasize that these are model-derived estimates rather than chronological ages, but we agree "predicted" is clearer and more appropriate. This change has been implemented throughout the manuscript for consistency and clarity.

14. Did you see the difference of the rejuvenation by eyes (visually)? Some pictures would be useful.

While we did collect baseline and day-30 photographs as part of standard documentation, visual assessment was not a predefined endpoint in our study design. The volar forearm surface typically shows subtle changes that, although corroborated with participant (undocumented) oral reports, are difficult to capture reliably in standard photography, particularly over a 30-day treatment period.

Without pre-specified clinical photography standards (standardized lighting, angles, and resolution), validated visual assessment scales, or blinded evaluators, including such images would not provide scientifically meaningful data.

15. Will the datasets be available? It would be very useful. The PRIDE ID is not available yet. "Sorry, no projects found for search term PXD062216 and the current set of active filters."

We thank the reviewer for the question. As far as we know, this is the largest proteomic skin dataset. The dataset is currently private in PRIDE under the ID PXD062216 and can be accessed using the reviewer login credentials (provided in the cover letter and in the manuscript's notes to the editor):

Username: reviewer_pxd062216@ebi.ac.uk

Password: DSipq6HbXnvL

In accordance with PRIDE's policy, the dataset will become publicly available upon publication of the manuscript.

Reviewer #3 (Remarks to the Author):

I co-reviewed this manuscript with one of the reviewers who provided the listed reports. This is part of the Communications Biology initiative to facilitate training in peer review and to provide appropriate recognition for Early Career Researchers who co-review manuscripts.

We commend Communications Biology's initiative to support Early Career Researchers in developing peer review expertise and thank you for your valuable contributions to improving our manuscript. The peer review process is indeed the bedrock of science where we collectively work to strengthen each other's research.

Reviewer #4 (Remarks to the Author):

Dear authors,

This study introduces a machine learning approach using Support Vector Regression (SVR) to predict biological skin age based on proteomic profiles. In a randomized, placebo-controlled trial involving 61 women aged 20–80, a 30-day topical treatment with quinoa bioester upregulated proteins associated with skin barrier function, antioxidant defense, and protease inhibition. The SVR model revealed that quinoa-treated skin appeared 11–16 years younger than placebo-treated skin in participants aged 50 and above. These findings provide a quantitative framework for evaluating anti-aging therapies and highlight quinoa bioester as a promising rejuvenating agent. However, I believe that some revisions and suggestions could further enhance the clarity, readability, and overall impact of the manuscript. These adjustments would help make the paper more accessible to a broader audience and better aligned with the standards of Communications Biology.

1. Please ensure consistency in terminology throughout the manuscript especially in the use of terms like SVR.

We thank the reviewer for this important observation. We have carefully reviewed the entire manuscript to ensure terminological consistency, particularly standardizing to "Support Vector Regression (SVR)" throughout, and have corrected all instances of inconsistent terminology.

Manuscript modifications for terminology consistency:

SVR terminology standardization:

- Before: "Support Vector Regressor (SVR)" After: "Support Vector Regression (SVR)" *Location: Title, Abstract, Introduction (first mention)*
- Before: "Support Vector Regressors (SVR)" After: "Support Vector Regression (SVR)" *Location: Methods section header*
- Before: "a Support Vector Regressor trained on" After: "Support Vector Regression trained on" *Location: Abstract, Results section*

Age terminology clarification:

- Before: "relative biological age" After: "predicted proteomic age" *Location: Throughout Results and Discussion sections*
- Before: "biological skin age" After: "proteomic age" *Location: Abstract and Introduction*

Other standardizations:

- Before: "mass spectrometry analysis" After: "mass spectrometry" *Location: Methods section*

2. I recommend briefly discussing the biological relevance of the quinoa bioester used in the SVR prediction, particularly in relation to its potential in vivo applications.

We thank the reviewer for this suggestion. We included the following text in the discussion:

“The observed reduction in predicted proteomic age following quinoa bioester treatment aligns with its known biological properties and suggests broader therapeutic potential. The SVR model's ability to detect these age-related shifts likely stems from quinoa bioester's multi-target action on key aging pathways. Rich in essential fatty acids (particularly linoleic and oleic acids) and phenolic compounds, quinoa bioester directly addresses multiple hallmarks of skin aging. The proteins driving the SVR age predictions, including those involved in barrier function (DSG1, DSC1, FLG), oxidative defense (SOD1, GLRX, GSS), and protease regulation (SERPINB3, SERPINB7), represent convergent pathways that quinoa bioester modulates simultaneously. This coordinated

action on multiple aging mechanisms, rather than targeting a single pathway, may explain the substantial age reduction observed in our model.

Our topical treatment results suggest potential for both preventive and therapeutic interventions. The more pronounced effects in older participants (≥50 years) indicate that quinoa bioester may be particularly effective in addressing accumulated age-related damage, possibly through its ability to restore compromised barrier function and reduce oxidative stress; both of which intensify with age. The 30-day treatment period demonstrated measurable proteomic shifts, suggesting that relatively short-term interventions can achieve molecular-level rejuvenation. Future applications could explore optimized delivery systems, combination therapies with other bioactives, or systemic formulations to extend these benefits beyond the treated skin areas. The fact that these changes are detectable through an unbiased machine learning approach strengthens the biological relevance of quinoa bioester as a legitimate anti-aging intervention rather than merely a cosmetic improvement."

3. Could you please clarify the source of the drawing and photograph in Figure 1? If the image was created by you or one of your co-authors, kindly cite the software used in the figure legend.

All drawings in the figures were created by the authors using Microsoft PowerPoint and BioRender (www.biorender.com). For the figures created with BioRender, we will include in the respective figure legends the statement: "Image created using Microsoft PowerPoint and BioRender".

4. Figure 3 description effectively explains the scatter plot and the use of the Leave-Age-Out (LAO) validation approach, which is appropriate for assessing age prediction performance. However, I recommend briefly clarifying why the LAO method was chosen over other cross-validation techniques, as this would help justify its use. Additionally, specifying the sample size (number of individuals) and providing confidence intervals for the MAE and R^2 would improve the robustness and interpretability of the model performance metrics. Lastly, consider stating whether any age-related bias or deviation from the y = x line was observed across the age spectrum.

We thank the reviewer for this constructive feedback.

1. Justification for LAO method: We chose Leave-Age-Out (LAO) validation, which is essentially leave-one-out cross-validation given our unique study design of one participant per age. Our principal motivation is that, even though from the proteomic perspective, as far as we know, this is the largest skin dataset to date, from a machine learning perspective, it is small. Moreover, with only one individual per age, traditional k-fold cross-validation would sacrifice too much of our training dataset.

2. Sample size clarification: We now explicitly state in the figure legend that n=61 individuals (one per age from 20-80).

As for confidence intervals and stating weather any age related bias or deviation from the y = x line was observed across the spectrum (explain this is complicated because the data is too noisy and challenging, we only observe a suggestive pattern).

5. In Figure 4, the left panel represents participants aged under 50, while the right panel represents participants aged 50 and older. Boxplots compare the predicted ages for bioactive-treated and placebo-treated arms," is generally clear but could be improved by specifying what the predicted age refers to (i.e., biological skin age) and whether the differences between groups are statistically significant. Including sample sizes and p-values in the figure legend or accompanying text would enhance interpretability and rigor.

We thank the reviewer for this suggestion. Albeit p-values were provided in the text, we recognize they should have also been in the legend for the sake of clarity. We have revised the Figure 4 legend to address all points raised:

Original Figure 4 Legend: "*Fig 4: Predicted Proteomic Age of Bioactive- and Placebo-Treated Skin, Stratified by Participant Age Group. The left panel represents participants aged under 50, while the right panel represents participants aged 50 and older. Boxplots compare the predicted ages for bioactive-treated and placebo-treated arms.*"

Revised Figure 4 Legend: "*Fig 4: Predicted Proteomic Age of Bioactive- and Placebo-Treated Skin, Stratified by Participant Age Group. The left panel represents participants aged under 50 (n=30), while the right panel represents participants aged 50 and older (n=31). Boxplots compare the predicted proteomic ages (derived from the SVR model trained on pre-treatment proteomic profiles) for bioactive-treated versus placebo-treated forearms from the same individuals after 30 days of treatment. Statistical significance was assessed using the Wilcoxon signed-rank test for paired comparisons: p=0.14 for the <50 group (median reduction of 11 years) and p<0.01 for the ≥50 group (median reduction of 16 years), comparing bioactive to placebo within each age stratum. The differences between age groups reflect the expected age-related changes in skin proteome, with older participants showing higher predicted proteomic ages in both treatment conditions.*"

The paired nature of our analysis is particularly important here, as each participant served as their own control with one forearm receiving bioactive treatment and the other receiving placebo, which strengthens the statistical power of our comparisons despite the stratification by age group.

6. Consider discussing whether the observed proteomic changes are expected to be transient or long-lasting, and whether continued application might enhance or diminish these effects over time. This is important for assessing the sustainability of the treatment's impact.

We thank the reviewer for this suggestion. As for now, we cannot make any evidence-based claims about effect persistence or long-term dynamics without additional data. We prefer not to speculate on temporal aspects in the manuscript. Nonetheless, we agree this represents an area for future investigation.

7. Were the proteomic responses consistent across different age groups (e.g., under 50 vs. over 50)? Since aging is a major factor in skin biology, discussing whether certain protein changes were more prominent in older participants would add valuable depth to the interpretation.

We appreciate the reviewer highlighting this important aspect, which we did address in our Results section and Figure 4. Our analysis revealed age-dependent responses, with participants ≥50 years showing significantly greater rejuvenation effects (16-year reduction in proteomic age, $p<0.01$) compared to younger participants (11-year reduction, $p=0.14$), as discussed in the section "Quinoa Bioester Treatment Lowers Predicted Proteomic Age." While individual protein-level stratification by age would indeed be interesting, our paired analysis design prioritized statistical power by analyzing the full cohort together, ensuring robust identification of key proteins (DSG1, DSC1, FLG, etc.). Moreover, we would need a bigger dataset to achieve this.

8. The discussion could be enhanced by synthesizing the observed changes into a unified mechanistic hypothesis or pathway-level interpretation for example, how quinoa bioester might coordinately regulate epidermal differentiation, oxidative defense, and barrier restoration.

We thank the reviewer for this valuable suggestion. We deliberately opted to maintain focus on our SVR-based methodology for unbiased age prediction as the primary contribution of this work. Our paired proteomic analysis identified key proteins across barrier function, oxidative defense, and protease regulation pathways, demonstrating coordinated effects across multiple skin aging mechanisms.

In our view, mechanistic pathway analyses are better conducted in controlled model systems where biological noise can be minimized. We previously performed extensive pathway-level analyses including GO enrichment using reconstructed human epidermis (Camillo-Andrade et al., Scientific Reports, 2020), where we comprehensively characterized the mechanistic pathways affected by quinoa bioester under controlled conditions. That paper motivated us to verify whether these beneficial effects could be detected in real-world human skin despite inherent biological variability from genetic diversity, environmental exposures, and inter-individual differences—which would make pathway enrichment analyses considerably noisier and less interpretable. Our success in detecting significant proteomic age reduction confirms that quinoa bioester's effects are

enough to manifest in vivo, providing essential validation that complements our previous mechanistic work.

Manuscript modification: To clarify this strategic choice, we have added one sentence to the Discussion section: "For comprehensive pathway enrichment analyses of quinoa bioester's mechanisms, we refer readers to our previous work in controlled reconstructed human epidermis **(Camillo-Andrade et al., 2020)**, as the current in vivo study prioritized validating these effects in real-world human skin through our unbiased SVR approach."

9. The reduction in predicted proteomic age (11–16 years) is significant and compelling. However, the manuscript could be strengthened by discussing how such changes compare to known clinical or phenotypic measures of rejuvenation. For instance, are there benchmarks or prior studies with similar anti-aging effects (even if non-proteomic) that can serve as context?

Indeed, we consider that citing other approaches and even presenting our results with a "grain of salt", considering the limitations of our work should be emphasized. We included this contextualization in the beginning of our discussion and are happy to have had time to step back and provide additional notice. Here is our newly included opening-discussion paragraph:

"To contextualize our proteomic findings, it is important to consider established benchmarks in anti-aging research, while acknowledging that different methods of evaluating aging, such as clinical parameters, epigenetic clocks, and proteomic profiles, represent fundamentally different measurement approaches, akin to using different rulers that may not be directly comparable. That said, topical interventions have been reported to yield modest effects over weeks; for example, vitamin C formulations have shown ~10–15% improvements in skin-aging parameters (DOI: 10.3390/antiox11081605), and tretinoin, to enhance collagen remodeling (DOI: 10.1056/NEJM199308193290803). Importantly, proteomic profiling of topical cosmeceutical efficacy has rarely been performed in humans; most studies have focused on pharmaceutical actives or irritants (DOI: 10.1371/journal.pone.0097772; 10.1038/s41598-023-37750-5), with cosmeceutical ingredients typically evaluated only in cell cultures and 3D skin models (DOI: 10.1038/s41598-020-76325-6). Systemic interventions using epigenetic clocks demonstrate larger but still modest effects; for example, metformin shows approximately

2-3 years of biological age deceleration over extended periods (DOI: 10.1016/j.cell.2024.08.021).

Our observed proteomic age shifts, although substantial, must be interpreted with caution given the inherently noisy nature of proteomic data from human skin samples, which show a high inter-individual variability. We emphasize that these numbers represent a suggestive trend, and our primary contribution is demonstrating that machine learning can detect treatment-associated proteomic patterns in an unbiased way, that is, without relying on a specific marker such as collagen. In addition, the use of a paired design, where treated regions were compared against control regions within the same participant before analysis across individuals, helped reduce variability, since part of the biological 'noise' was normalized internally. Nevertheless, considering this caveat along with other limitations of the study, such as having only one participant per age, we recommend that our numerical predictions eventually be tested against larger and more diverse populations.

10. Although the study was conducted in women, and menopause is mentioned, it would be valuable to discuss whether hormonal status was assessed or controlled for, and whether the findings may translate to males or postmenopausal populations beyond age grouping. How might these findings apply to individuals with different skin types, and ethnic backgrounds?

We thank the reviewer for highlighting these important considerations regarding study generalizability. We did not assess individual hormonal status, instead using age 50 as a pragmatic cutoff to approximate pre- and post-menopausal groups, which we acknowledge as a limitation. Our study cohort was intentionally restricted to female participants with Fitzpatrick skin types II-IV from a single geographic location to minimize variability due to experimental feasibility and budget. We fully agree that extending this work to include males, diverse ethnic backgrounds, and different skin phototypes represents critical next steps, but we deliberately avoided speculating about generalizability without supporting data. We are seeking funds to expand this study.

11. The manuscript notably lacks a conclusion section, which is essential for summarizing the key findings, reinforcing the scientific contributions, and outlining the broader implications of the work.

In order to address this suggestion, we included a concluding paragraph at the end of the manuscript. We reflected it below for your convenience.

Conclusion

"*This study presents an unbiased machine learning framework using Support Vector Regression to assess whether proteomic profiles can identify younger molecular signatures in skin following cosmeceutical treatment. To our knowledge, we report the largest skin proteomic dataset to date (244 samples from 61 participants, analyzed in technical duplicates). Our data demonstrated that 30-day topical treatment with quinoa bioester induced measurable shifts in the skin proteome, with treated samples showing predicted proteomic ages younger than placebo-treated controls, particularly in participants aged 50 and above. The paired proteomic analysis revealed coordinated changes in proteins governing skin barrier function (DSG1, DSC1, FLG), oxidative defense (SOD1, GLRX, GSS), and protease regulation (SERPINB3, SERPINB7), consistent with mechanisms of skin rejuvenation. While our findings are limited to female participants with Fitzpatrick skin types II-IV and require validation in more diverse populations, this work establishes a quantitative framework for evaluating anti-aging interventions that moves beyond subjective assessments. Future studies should explore treatment persistence, dose-response relationships, and extend to broader demographic groups. Despite inherent challenges in modeling complex biological aging processes, this proof-of-concept demonstrates that machine learning approaches can detect treatment-induced proteomic shifts in human skin, providing a foundation for objective assessment of cosmeceutical efficacy and opening new avenues for evidence-based and unbiased evaluation of skin health interventions.*"

12. A few additional experiments or analyses could further strengthen the scientific rigor and impact of the study, particularly given the standards of Communications Biology. (i) Exploring different concentrations of quinoa bioester or sampling at multiple time points during treatment could reveal the dynamics and optimal conditions of its effect on skin proteomics. (ii) Conducting a systems-level analysis (e.g., gene ontology or pathway enrichment analysis) of the differentially abundant proteins would provide mechanistic insights and strengthen the biological interpretation of the findings.

While dose-response and temporal dynamics studies would indeed be valuable, implementing these would require an entirely new clinical trial. Our current study involved 61 participants and we took over 4 years with 244 proteomic samples, representing substantial investment of resources and participant commitment. Adding concentration variations and multiple timepoints would multiply this complexity several-fold, plus requiring funding for personnel of which we don't currently dispose.

As noted in our response to point 8, we previously conducted comprehensive pathway enrichment analyses using quinoa bioester in controlled reconstructed human epidermis (Camillo-Andrade et al., Scientific Reports, 2020). The current study was specifically designed to validate whether these mechanistic effects translate to real-world human skin. Repeating GO analysis on this inherently noisier in vivo dataset would yield less

interpretable results than our previous controlled study, which readers can reference for detailed mechanistic insights.

13. The manuscript does not exhibit any major technical or conceptual flaws that would prohibit its publication. The study is methodologically sound and presents an innovative approach using support vector regression (SVR) on proteomic data to estimate biological skin age and evaluate the rejuvenating potential of quinoa bioester.

We are greatly encouraged by Reviewer #4's concluding remarks that the manuscript is "methodologically sound" and does not exhibit "any major technical or conceptual flaws" is deeply appreciated. We thank the reviewer once again. We firmly believe that the revisions undertaken have further strengthened the work.

Dear Dr., Bircan,

We thank you and the reviewers for reviewing our manuscript and appreciate the constructive feedback and the opportunity to strengthen our work through revision. We are encouraged that the reviewers found our integration of proteomics with machine learning to be novel and of interest; in particular, Reviewer #4 noted no major technical or conceptual flaws that would prohibit publication. We have carefully considered all comments and have substantially revised the manuscript to address the constructive suggestions. Please find below each point raised by the reviewers together with the actions taken and justifications.

Reviewer #1 (Remarks to the Author):

The manuscript "Quinoa Bioester Treatment Shifts Human Skin Proteome Toward Younger Molecular Signatures" by Camillo-Andrade et al. describes a machine learning approach using Support Vector Regression to predict biological skin age from proteomic profiles, enabling objective assessment of anti-aging interventions. The integration of proteomics with machine learning represents a novel and unbiased method for evaluating treatment effectiveness, and the findings suggest that quinoa bioester treatment can shift molecular signatures toward a younger state.

We sincerely thank the reviewer for recognizing the novelty of our unbiased approach and for the insightful understanding that this provides a framework for evaluating topical treatments.

Several experiments, however, need further clarification. Aging is a complex process that involves changes not only at the proteomic level, but also at the genomic, epigenetic, and other molecular levels. In the context of skin aging, morphological changes should also be considered alongside molecular alterations.

Does this correlate with any visible or clinical improvement in skin condition?

We agree that aging encompasses multiple molecular levels beyond proteomics. We did observe visible improvements that correlated with our proteomic findings. Besides, participants independently reported noticeable improvements in skin texture and hydration, albeit claiming in both arms, which, as noted in our manuscript, the placebo also contained hydrating base ingredients.

Could the authors also characterize the treatment effectiveness by clinical scales for measuring skin aging?

We thank the reviewer for this suggestion. Although classical clinical aging scales were not part of our design, we conducted quantitative skin hydration assessments using the SkinUp®, a digital analyzer based on bioelectrical impedance. Increased hydration reduces skin impedance by enabling more continuous conductive pathways formed by

water and ions. The device provides relative hydration values in arbitrary units and has been validated in vivo against the gold-standard Corneometer® CM825 (DOI: 10.1111/srt.12833). Higher values indicate greater hydration.

Briefly, skin hydration measurements in all 61 participants showed that quinoa bioester treatment produced greater improvements compared to placebo (mean Δ = 1.14 for treated vs 0.54 for placebo, *p*-value = 0.17), representing a 111% greater hydration improvement, directionally consistent with proteomic findings effect size. This data is not in the original manuscript.

It is important to note that our p-value of 0.17 exceeds the conventional 0.05 threshold; nonetheless, after reflecting in the light of the reviewer's comments, we believe these data indeed merit inclusion in our manuscript. Although not statistically significant, a p-value of 0.17 suggests a trend that would occur by chance alone in less than, roughly, one in five times, and when combined with a substantial effect size (111% greater improvement: mean Δ = 1.14 vs 0.54 arbitrary units), provides suggestive evidence worthy of report. The consistent directional improvement across participants, coupled with our statistically significant proteomic findings, provides multi-level support for the treatment's beneficial effects. We have included these hydration measurements as Supplementary Table 1 and integrated discussion of these findings into the manuscript.

Manuscript modifications made:

1. Methods section addition (Sample Collection subsection): Added after the treatment protocol description*:* "Skin hydration was quantitatively assessed using the SkinUp® digital analyzer, which measures bioelectrical impedance. This device has been validated against the gold-standard Corneometer® CM825 (DOI: 10.1111/srt.12833). Measurements were taken from the same standardized forearm areas before and after the 30-day treatment period."

2. Results section addition (new paragraph after the paired proteomic analysis): Added: "To complement our proteomic findings, we assessed skin hydration changes using bioelectrical impedance measurements. Hydration analysis across all 61 participants revealed that both the placebo and the quinoa bioester treatment produced improvements in skin hydration (mean Δ = 0.54 arbitrary units for placebo vs 1.14 for bioactive, p = 0.17), with the quinoa bioester group showing improvement compared to placebo. While this difference did not reach conventional statistical significance ($p > 0.05$), the consistent directional improvement suggests a meaningful biological effect. The high inter-individual variability expected given our diverse age range (20-80 years), combined with the hydrating effects of the placebo base formulation itself, makes detecting additional bioactive-specific effects particularly challenging through conventional metrics alone. Skin hydration measurement data are available in Supplementary File 4."

3. Discussion section modification: Added to the paragraph discussing treatment effects: "The proteomic age reduction observed with quinoa bioester treatment was supported by quantitative hydration measurements, which showed a 111% greater

improvement compared to placebo. This multi-level evidence—combining molecular signatures with functional skin parameters corroborates to our conclusion that quinoa bioester provides measurable skin health benefits."

Additionally, while the treatment duration was 30 days—are there data on how long the molecular age shift persists after stopping treatment?

We appreciate this important suggestion; however, our study did not include post-treatment follow-up. Such an extension would require additional skin sampling from the same participants at multiple timepoints, which presents practical challenges with volunteer retention and increases participant burden through repeated skin collection procedures. Nonetheless, we fully agree that investigating the persistence of these proteomic changes represents an important direction for future research.

Reviewer #2 (Remarks to the Author):

This study developed a proteomics aging clock for human skin and concluded that quinoa bioester treatment shifts the human skin proteome toward younger states. It is a very interesting study, however, we have some concerns, and questions, detailed, below.

We thank the reviewer for their detailed technical assessment and thoughtful questions. These important points have helped us clarify our methodology and strengthen the manuscript. We address each concern below:

1. Fig. 4 shows that the median predicted proteomic age of the post-treatment samples much higher than the median chronological age of the placebo groups and the young bioactive group. Does it make sense that the placebo aged the skin?

We appreciate the reviewer's observation. The systematic elevation in predicted ages following any topical treatment, including placebo, reflects a domain shift phenomenon in machine learning, where models encounter distributions outside their training space. Our model, trained exclusively on untreated skin, detects the proteomic alterations induced by 30 days of twice-daily application and interprets these through its learned framework. Importantly, while absolute predictions shift upward, the preserved relative difference between treatments demonstrates the model's discriminative capacity remains intact; this is a key finding. Our choice of a single linear SVR, while unable to capture all age-related non-linearities partially visible in Figure 3, was deliberate to avoid overfitting given our sample size constraints. We fully agree with the reviewer that these elevated predictions warrant discussion; nonetheless, we interpret them as the model successfully detecting that treated skin differs from untreated skin while maintaining the ability to discriminate treatment efficacy on completely new samples. As now better reflected in the revised manuscript, given several points raised by this reviewer that should be made more evident in our work, our primary contribution is demonstrating differential treatment effects rather than precise absolute age estimation in treated skin.

We rather think that the proteomic aging clock highly overfit as it performed much worse in the unseen samples. Maybe a more straightforward and usual method would be better (e.g., glmnet as it contain a built-in parameter optimization; it selects the important features too).

The model maintains predictive relationships in post-treatment data; the correlation between predicted ages for bioactive versus placebo remains consistent, with bioactive samples systematically predicted several years younger. As such, we presume that what the reviewer mentioned as poor performance is the systematic offset discussed above, not degraded predictive capability. This distinction is crucial: overfitting produces erratic, unreliable predictions on new data, whereas our model shows consistent, interpretable shifts that preserve treatment discrimination in never seen data.

The elevated ages reflect a well-documented phenomenon in machine learning, domain shift, where models encounter data from a different distribution than their training set. Our model was trained only on untreated skin. Importantly, the model's ability to discriminate between treatments remains robust, which is our primary objective.

Regarding methodology, we actually started this project with Lasso regression / elastic net, exactly as suggest; we were motivated by glmnet's elegant built-in cross-validation and feature selection. We spent considerable time with these approaches (as they were our initial preference). However, when we compared performance head-to-head, linear SVR-RFE, we gained an edged, albeit marginally. In our specific context with high-dimensional proteomic data, SVR's margin-based optimization seemed to handle the noise slightly better.

That said, the reviewer is absolutely right that both are linear methods with different flavors of regularization; yet, switching between them did not resolve the domain shift we're seeing. We chose to present just the SVR results to keep the narrative clean, but we completely understand why the glmnet is an interesting option.

2. It may be problematic to train and test the models on the same individuals (same forearm pre-treatment and post-treatment). Usually, samples of a given subject are not recommended for use in both the training and testing sets.

We appreciate the reviewer raising this important methodological consideration. We acknowledge the point of using samples from the same individuals in both training and testing phases, where independent datasets are preferred. However, we respectfully argue that our approach is both intentional and appropriate for our specific research question, which fundamentally differs from developing a deployable age prediction model.

Our group's experience with proteomic studies in human subjects has consistently demonstrated that inter-individual variability far exceeds intra-individual changes, even after interventional treatments (Brunoro et al., BMC Cancer 2019; Camillo-Andrade et al., Sci Data 2024); these manuscripts are referenced in the main text. Unlike controlled cell

culture experiments, human skin proteomics exhibits enormous biological variability driven by genetics, lifestyle, environmental exposures, and unmeasured factors. This variability is further amplified after a 30-day treatment period, where individual responses to topical formulations vary substantially based on skin barrier function, absorption rates, and metabolic differences.

Critically, our research question is not "Can we build a generalizable age predictor for any new individual?", in which the reviewer would be completely right to be concerned; but rather "Can quinoa bioester treatment induce detectable rejuvenation-associated proteomic shifts within the same individuals?" This distinction is critical and fundamental. We are using the SVR model as an analytical tool to detect within-subject treatment effects, not as a predictive model for deployment on independent populations. The paired design, where each individual serves as their own control, is essential for this purpose, as it allows us to:

1. Control for the massive inter-individual proteomic variation that would otherwise overwhelm treatment signals
2. Detect subtle but consistent treatment-induced changes that manifest differently across individuals due to baseline heterogeneity
3. Ensure that observed differences reflect treatment effects rather than population stratification or batch effects

If we had used completely independent individuals for training and testing, we would be answering a different question entirely: whether age patterns learned from one population can predict ages in another population after treatment. This would conflate two sources of variation (inter-individual differences and treatment effects) making it way harder to isolate the treatment's impact and requiring much larger cohorts which would exceed our budget by far.

We note that the 30-day temporal gap and the domain shift observed (elevated predicted ages in all treated samples) actually demonstrate that our model is not simply memorizing individual patterns. If it were, we would expect accurate age predictions post-treatment. Instead, the systematic offset indicates the model correctly identifies that treated skin differs proteomically from untreated skin, while still maintaining the ability to discriminate between treatment conditions.

We thank the reviewer for raising this important methodological point, which is a legit and fundamental consideration. This critical observation should be made evident in the main text, we now added a paragraph to the Discussion.

Manuscript modification for Discussion section: "An important consideration in our study design is the use of samples from the same individuals for both model training (pre-treatment) and testing (post-treatment). Such experimental design is motivated for detecting within-subject treatment effects rather than building a generalizable age predictor for new populations. The massive inter-individual variability in human skin proteomics, which far exceeds treatment-induced changes, necessitates paired analysis to better focus on treatment effects. We emphasize that our SVR model serves as an analytical tool for quantifying relative proteomic shifts within individuals, and not as a

deployable age prediction system. Future studies seeking to develop generalizable proteomic age clocks would require independent and unpaired validation cohorts.

3. It seems that the clock predicted very large skin age (140 years in some cases) for the placebo (and even some bioactive) samples, but not for the pre-treatment samples. Have the authors checked the possible reasons/mechanisms behind this phenomenon?

We attribute the elevated predictions to domain shift, where our model trained on untreated skin must extrapolate when encountering treated skin. Bioactive-treated samples consistently show younger predicted ages than placebo-treated samples. We could indeed use more sophisticated regressors, such as, say, a fitting a sigmoid with asymptotic behavior at the training dataset min and max, but decided to stay with the linear regression and all of its limitations in exchange for diminishing overfitting risks.

4. It would be useful to see performance metrics for the post-treatment samples too (placebo and bioactive samples separately).

We intentionally present box plots rather than traditional performance metrics (MAE, R^2) as these metrics assume accurate absolute age prediction, which is not our objective. The systematic domain shift affects both placebo and bioactive treatments equally—they were applied identically for 30 days, creating the same methodological conditions. What matters is that despite this uniform shift, bioactive samples are consistently predicted 11-16 years younger than their paired placebo controls ($p<0.01$ for ≥50 group). This differential is observed across 61 individuals, shows age-dependent magnitude (stronger in older participants where aging effects are more pronounced), and aligns with our mechanistic findings of enhanced barrier proteins and reduced oxidative stress markers. The reproducibility of this relative difference under identical domain shift conditions demonstrates genuine biological signal rather than random variation.

Here we authors joined the reviewer's question's 5 and 7 below as we believe our response addresses both questions, jointly.

5. It would be also useful to explore the most important features in the model to see which mechanisms of aging the clock captures. How much do these proteins overlap with the differentially expressed proteins? 7. It would be interesting to see how many proteins remained after feature elimination. A large number of proteins are necessary to explain the aging process or a few is enough to capture it?

This is an interesting point as we were also curious if there were a minimal set of which we could predict aging. It turns out that we were able to converge to an RFE-SVR panel of 200 without losing MAE performance, that remains stable in MAE until 120 proteins. Five proteins overlap with our paired comparison panel that follows treatment. This limited overlap is somewhat expected and the proteins are biologically meaningful in regards to aging. The SVR panel identifies proteins whose baseline levels correlate with chronological age (trained on untreated samples), while paired analysis identifies acute

treatment responses and is derived from proteomes pos-treatment. We noted that such overlapping proteins are established hallmarks of aging**:**

- **Thioredoxin**: Master antioxidant that declines with age; its upregulation suggests enhanced oxidative stress defense, a key mechanism of rejuvenation (DOI: 10.1016/j.ejphar.2022.174756 / DOI: 10.3390/antiox12051078.)
- **Kallikrein-7**: Serine protease controlling desquamation; dysregulated in aged skin causing impaired barrier function (DOI: 10.1097/JD9.0000000000000036)
- **Vinculin**: Mechanotransduction protein linking cellular adhesion to cytoskeleton; altered expression affects tissue integrity and wound healing in aging (DOI: 10.1007/s00018-017-2511-3/ DOI: 10.1083/jcb.122.6.1285).
- **Myosin**: Non-muscle myosin essential for cellular mechanics; changes affect fibroblast contractility and ECM remodeling (DOI: 10.1016/j.yexcr.2014.04.013).
- **Ganglioside GM2 activator** is associated with aging because its altered expression in aged skin has been linked to lysosomal dysfunction and impaired lipid metabolism, processes that contribute to age-related skin barrier decline (DOI: 10.1016/j.nbd.2025.106798)

6. It would be useful to illustrate in a figure the abundance change of proteins revealed by the paired proteomic analysis to support the findings.

We now highlighted that the fold changes are also available in Supplementary Table 3 in the manuscript's main text and thank the reviewer for pointing this out. As for the Figure, we prefer to maintain focus on our primary contribution, the unbiased SVR-based prediction. As such, we modified the manuscript as follows:

Manuscript alterations

Original text (end of "Paired Proteomic Analysis Reveals..." section): "A complete list of differentially abundant proteins is available in Supplementary File 3."

Modified text: "A complete list of differentially abundant proteins, including Log_2 fold changes and *p*-values identified in the paired analysis between quinoa bioester and placebo treatments, is available in Supplementary File 3. "

8. Showing the predictions paired in Fig. 4.(by using paired box plots between the treated and placebo samples would provide additional, useful information. Also, what is the number of samples in each groups?

We thank the reviewer for this suggestion. While we considered paired plots to show individual responses, we prefer to maintain separate box plots as they are simpler to interpret, emphasize population-level effects, and avoid visual clutter with 30-31 connecting lines per panel. However, we have revised the Figure 4 legend to clearly specify the sample sizes and paired nature of the data: n=30 individuals aged 20-49 years and n=31 individuals aged 50-80 years, with each participant contributing both bioactive and placebo measurements. We emphasize that although the visualization uses separate box plots, our statistical analysis appropriately employed the Wilcoxon signed-rank test for paired comparisons, accounting for the within-subject design where each participant served as their own control.

Original Fig 4 Legend: Predicted Proteomic Age of Bioactive- and Placebo-Treated Skin, Stratified by Participant Age Group. The left panel represents participants aged under 50, while the right panel represents participants aged 50 and older. Boxplots compare the predicted ages for bioactive-treated and placebo-treated arms.

Revised Figure 4 Legend: "Fig 4: Predicted Proteomic Age of Bioactive- and Placebo-Treated Skin, Stratified by Participant Age Group. The left panel represents participants aged under 50 (n=30), while the right panel represents participants aged 50 and older (n=31). Boxplots compare the predicted proteomic ages (derived from the SVR model trained on pre-treatment proteomic profiles) for bioactive-treated versus placebo-treated forearms from the same individuals after 30 days of treatment. Statistical significance was assessed using the Wilcoxon signed-rank test for paired comparisons: $p=0.14$ for the <50 group (median reduction of 11 years) and $p<0.01$ for the ≥50 group (median reduction of 16 years), comparing bioactive to placebo within each age stratum. The differences between age groups reflect the expected age-related changes in skin proteome, with older participants showing higher predicted proteomic ages in both treatment conditions*".*

9. How was the hyperparameter optimization done? A clear description is needed. Which samples (how many) was used for the optimization, which parameters where used, which was the best? The same is true for the feature selection procedure.

One advantage of linear SVR is that it contains only the regularization parameter C, thus requiring minimal hyperparameter tuning. We tested C values of [10, 100, 1000, 10000, 100000] using Leave-Age-Out cross-validation and selected C=100000 based on minimum MAE. Details have been added to the Methods section.

Manuscript modification (Methods - SVR subsection):

Revised text: "We performed Leave-Age-Out cross-validation on pre-treatment samples (n=122) to simultaneously optimize hyperparameters and evaluate model performance. Testing regularization parameter C values of [10, 100, 1000, 10000, 100000], we selected C=100000 which achieved MAE = 7.17 years ($R^2 = 0.74$).

10. Was the min-max scaling done separately on the training and post-treatment data to avoid information leakage?

The scaler was fitted only on the training data, and these same scaling parameters were then applied to transform the post-treatment samples.

To make this more evident, we revised the manuscript as follows:

Current text (in Methods - RFE section): "Protein quantitation values were normalized to a 0-1 range using MinMax scaling to ensure all features contributed equally to the model regardless of their original scale."

Revised text with addition: "Protein quantitation values were normalized to a 0-1 range using MinMax scaling to ensure all features contributed equally to the model regardless of their original scale. The scaler was fitted exclusively on the pre-treatment training samples, and these same scaling parameters were subsequently applied to transform the post-treatment test samples, ensuring consistent normalization without incorporating any information from the test set distribution. All feature selection (RFE) were performed within a nested LAO framework; in each fold, feature selection and hyperparameter optimization were fit on the training ages only, then evaluated on the held-out age."

11. In line 362: How did the authors find exactly one patient for each year of age?

Thank you for this question. This was indeed a challenging requirement, which we anticipated and addressed through the study design. We used age-stratified quota sampling with single-year strata across the targeted age range. Recruitment was conducted via public calls for volunteers, after which respondents completed a standardized screening form. A key inclusion rule was uniqueness of year of birth: we verified year of birth using official identification and enrolled only one individual per birth-year stratum. Enrollment proceeded iteratively with continuous monitoring of the accrual log; once a stratum was filled, subsequent candidates from the same birth year were kept as alternates. If an enrolled participant withdrew before baseline assessment, the next eligible alternate from the same stratum was contacted, thereby preserving the one-per-year structure. Age was defined as completed years at the baseline (index) visit to avoid misclassification from rounding. This preplanned, criterion-based procedure ensured exactly one participant for each year of age in the final sample.

Manuscript modifications made:

1. **Methods section addition (Sample Collection subsection):**

Age-stratified enrollment. We prospectively used single-year quota sampling across the prespecified 20–80 range. Volunteers were openly recruited and screened with a standardized form, ID verification, and considering year of birth; we enrolled one

participant per birth-year. Age was defined as completed years at baseline, ensuring one participant of each age.

12. in line 373: How did the authors make sure that participants did not switch the formula? Were the containers labeled such as “for left arm” or “for right arm”?

As the reviewer correctly presumed, the containers were clearly labeled "right forearm" or "left forearm" with accompanying written instructions. Additionally, we strategically chose the volar forearm site as, among other advantages such as low sun exposure, it inherently minimizes cross-contamination risk as participants necessarily need to use different hands to apply each product.

Manuscript modification: *Added to Methods section after randomization statement:* "Formulation tubes were labeled 'right forearm' or 'left forearm' with accompanying written instructions to ensure correct application throughout the treatment period."

13. in line 470: What is "relative" biological age? Why relative?

We thank the reviewer for identifying this confusing terminology. We have revised the manuscript to consistently use "predicted biological age" throughout, which more accurately describes what our SVR model outputs—a prediction of biological age based on proteomic profiles. Even though we now make more evident that our model is not an age predictor in the manuscript but used to compare the effect of the treatments. The term "relative" was initially used to emphasize that these are model-derived estimates rather than chronological ages, but we agree "predicted" is clearer and more appropriate. This change has been implemented throughout the manuscript for consistency and clarity.

14. Did you see the difference of the rejuvenation by eyes (visually)? Some pictures would be useful.

While we did collect baseline and day-30 photographs as part of standard documentation, visual assessment was not a predefined endpoint in our study design. The volar forearm surface typically shows subtle changes that, although corroborated with participant (undocumented) oral reports, are difficult to capture reliably in standard photography, particularly over a 30-day treatment period.

Without pre-specified clinical photography standards (standardized lighting, angles, and resolution), validated visual assessment scales, or blinded evaluators, including such images would not provide scientifically meaningful data.

15. Will the datasets be available? It would be very useful. The PRIDE ID is not available yet. "Sorry, no projects found for search term PXD062216 and the current set of active filters."

We thank the reviewer for the question. As far as we know, this is the largest proteomic skin dataset. The dataset is currently private in PRIDE under the ID PXD062216 and can be accessed using the reviewer login credentials (provided in the cover letter and in the manuscript's notes to the editor):

Username: reviewer_pxd062216@ebi.ac.uk

Password: DSipq6HbXnvL

In accordance with PRIDE's policy, the dataset will become publicly available upon publication of the manuscript.

Reviewer #3 (Remarks to the Author):

I co-reviewed this manuscript with one of the reviewers who provided the listed reports. This is part of the Communications Biology initiative to facilitate training in peer review and to provide appropriate recognition for Early Career Researchers who co-review manuscripts.

We commend Communications Biology's initiative to support Early Career Researchers in developing peer review expertise and thank you for your valuable contributions to improving our manuscript. The peer review process is indeed the bedrock of science where we collectively work to strengthen each other's research.

Reviewer #4 (Remarks to the Author):

Dear authors,

This study introduces a machine learning approach using Support Vector Regression (SVR) to predict biological skin age based on proteomic profiles. In a randomized, placebo-controlled trial involving 61 women aged 20–80, a 30-day topical treatment with quinoa bioester upregulated proteins associated with skin barrier function, antioxidant defense, and protease inhibition. The SVR model revealed that quinoa-treated skin appeared 11–16 years younger than placebo-treated skin in participants aged 50 and above. These findings provide a quantitative framework for evaluating anti-aging therapies and highlight quinoa bioester as a promising rejuvenating agent. However, I believe that some revisions and suggestions could further enhance the clarity, readability, and overall impact of the manuscript. These adjustments would help make the paper more accessible to a broader audience and better aligned with the standards of Communications Biology.

1. Please ensure consistency in terminology throughout the manuscript especially in the use of terms like SVR.

We thank the reviewer for this important observation. We have carefully reviewed the entire manuscript to ensure terminological consistency, particularly standardizing to "Support Vector Regression (SVR)" throughout, and have corrected all instances of inconsistent terminology.

Manuscript modifications for terminology consistency:

SVR terminology standardization:

- Before: "Support Vector Regressor (SVR)" After: "Support Vector Regression (SVR)" *Location: Title, Abstract, Introduction (first mention)*
- Before: "Support Vector Regressors (SVR)" After: "Support Vector Regression (SVR)" *Location: Methods section header*
- Before: "a Support Vector Regressor trained on" After: "Support Vector Regression trained on" *Location: Abstract, Results section*

Age terminology clarification:

- Before: "relative biological age" After: "predicted proteomic age" *Location: Throughout Results and Discussion sections*
- Before: "biological skin age" After: "proteomic age" *Location: Abstract and Introduction*

Other standardizations:

- Before: "mass spectrometry analysis" After: "mass spectrometry" *Location: Methods section*

2. I recommend briefly discussing the biological relevance of the quinoa bioester used in the SVR prediction, particularly in relation to its potential in vivo applications.

We thank the reviewer for this suggestion. We included the following text in the discussion:

“The observed reduction in predicted proteomic age following quinoa bioester treatment aligns with its known biological properties and suggests broader therapeutic potential. The SVR model's ability to detect these age-related shifts likely stems from quinoa bioester's multi-target action on key aging pathways. Rich in essential fatty acids (particularly linoleic and oleic acids) and phenolic compounds, quinoa bioester directly addresses multiple hallmarks of skin aging. The proteins driving the SVR age predictions, including those involved in barrier function (DSG1, DSC1, FLG), oxidative defense (SOD1, GLRX, GSS), and protease regulation (SERPINB3, SERPINB7), represent convergent pathways that quinoa bioester modulates simultaneously. This coordinated

action on multiple aging mechanisms, rather than targeting a single pathway, may explain the substantial age reduction observed in our model.

Our topical treatment results suggest potential for both preventive and therapeutic interventions. The more pronounced effects in older participants (≥50 years) indicate that quinoa bioester may be particularly effective in addressing accumulated age-related damage, possibly through its ability to restore compromised barrier function and reduce oxidative stress; both of which intensify with age. The 30-day treatment period demonstrated measurable proteomic shifts, suggesting that relatively short-term interventions can achieve molecular-level rejuvenation. Future applications could explore optimized delivery systems, combination therapies with other bioactives, or systemic formulations to extend these benefits beyond the treated skin areas. The fact that these changes are detectable through an unbiased machine learning approach strengthens the biological relevance of quinoa bioester as a legitimate anti-aging intervention rather than merely a cosmetic improvement."

3. Could you please clarify the source of the drawing and photograph in Figure 1? If the image was created by you or one of your co-authors, kindly cite the software used in the figure legend.

All drawings in the figures were created by the authors using Microsoft PowerPoint and BioRender (www.biorender.com). For the figures created with BioRender, we will include in the respective figure legends the statement: "Image created using Microsoft PowerPoint and BioRender".

4. Figure 3 description effectively explains the scatter plot and the use of the Leave-Age-Out (LAO) validation approach, which is appropriate for assessing age prediction performance. However, I recommend briefly clarifying why the LAO method was chosen over other cross-validation techniques, as this would help justify its use. Additionally, specifying the sample size (number of individuals) and providing confidence intervals for the MAE and R^2 would improve the robustness and interpretability of the model performance metrics. Lastly, consider stating whether any age-related bias or deviation from the y = x line was observed across the age spectrum.

We thank the reviewer for this constructive feedback.

1. Justification for LAO method: We chose Leave-Age-Out (LAO) validation, which is essentially leave-one-out cross-validation given our unique study design of one participant per age. Our principal motivation is that, even though from the proteomic perspective, as far as we know, this is the largest skin dataset to date, from a machine learning perspective, it is small. Moreover, with only one individual per age, traditional k-fold cross-validation would sacrifice too much of our training dataset.

2. Sample size clarification: We now explicitly state in the figure legend that n=61 individuals (one per age from 20-80).

As for confidence intervals and stating weather any age related bias or deviation from the y = x line was observed across the spectrum (explain this is complicated because the data is too noisy and challenging, we only observe a suggestive pattern).

5. In Figure 4, the left panel represents participants aged under 50, while the right panel represents participants aged 50 and older. Boxplots compare the predicted ages for bioactive-treated and placebo-treated arms," is generally clear but could be improved by specifying what the predicted age refers to (i.e., biological skin age) and whether the differences between groups are statistically significant. Including sample sizes and p-values in the figure legend or accompanying text would enhance interpretability and rigor.

We thank the reviewer for this suggestion. Albeit p-values were provided in the text, we recognize they should have also been in the legend for the sake of clarity. We have revised the Figure 4 legend to address all points raised:

Original Figure 4 Legend: "*Fig 4: Predicted Proteomic Age of Bioactive- and Placebo-Treated Skin, Stratified by Participant Age Group. The left panel represents participants aged under 50, while the right panel represents participants aged 50 and older. Boxplots compare the predicted ages for bioactive-treated and placebo-treated arms.*"

Revised Figure 4 Legend: "*Fig 4: Predicted Proteomic Age of Bioactive- and Placebo-Treated Skin, Stratified by Participant Age Group. The left panel represents participants aged under 50 (n=30), while the right panel represents participants aged 50 and older (n=31). Boxplots compare the predicted proteomic ages (derived from the SVR model trained on pre-treatment proteomic profiles) for bioactive-treated versus placebo-treated forearms from the same individuals after 30 days of treatment. Statistical significance was assessed using the Wilcoxon signed-rank test for paired comparisons: $p=0.14$ for the <50 group (median reduction of 11 years) and $p<0.01$ for the ≥50 group (median reduction of 16 years), comparing bioactive to placebo within each age stratum. The differences between age groups reflect the expected age-related changes in skin proteome, with older participants showing higher predicted proteomic ages in both treatment conditions.*"

The paired nature of our analysis is particularly important here, as each participant served as their own control with one forearm receiving bioactive treatment and the other receiving placebo, which strengthens the statistical power of our comparisons despite the stratification by age group.

6. Consider discussing whether the observed proteomic changes are expected to be transient or long-lasting, and whether continued application might enhance or diminish these effects over time. This is important for assessing the sustainability of the treatment's impact.

We thank the reviewer for this suggestion. As for now, we cannot make any evidence-based claims about effect persistence or long-term dynamics without additional data. We prefer not to speculate on temporal aspects in the manuscript. Nonetheless, we agree this represents an area for future investigation.

7. Were the proteomic responses consistent across different age groups (e.g., under 50 vs. over 50)? Since aging is a major factor in skin biology, discussing whether certain protein changes were more prominent in older participants would add valuable depth to the interpretation.

We appreciate the reviewer highlighting this important aspect, which we did address in our Results section and Figure 4. Our analysis revealed age-dependent responses, with participants ≥50 years showing significantly greater rejuvenation effects (16-year reduction in proteomic age, $p<0.01$) compared to younger participants (11-year reduction, $p=0.14$), as discussed in the section "Quinoa Bioester Treatment Lowers Predicted Proteomic Age." While individual protein-level stratification by age would indeed be interesting, our paired analysis design prioritized statistical power by analyzing the full cohort together, ensuring robust identification of key proteins (DSG1, DSC1, FLG, etc.). Moreover, we would need a bigger dataset to achieve this.

8. The discussion could be enhanced by synthesizing the observed changes into a unified mechanistic hypothesis or pathway-level interpretation for example, how quinoa bioester might coordinately regulate epidermal differentiation, oxidative defense, and barrier restoration.

We thank the reviewer for this valuable suggestion. We deliberately opted to maintain focus on our SVR-based methodology for unbiased age prediction as the primary contribution of this work. Our paired proteomic analysis identified key proteins across barrier function, oxidative defense, and protease regulation pathways, demonstrating coordinated effects across multiple skin aging mechanisms.

In our view, mechanistic pathway analyses are better conducted in controlled model systems where biological noise can be minimized. We previously performed extensive pathway-level analyses including GO enrichment using reconstructed human epidermis (Camillo-Andrade et al., Scientific Reports, 2020), where we comprehensively characterized the mechanistic pathways affected by quinoa bioester under controlled conditions. That paper motivated us to verify whether these beneficial effects could be detected in real-world human skin despite inherent biological variability from genetic diversity, environmental exposures, and inter-individual differences—which would make pathway enrichment analyses considerably noisier and less interpretable. Our success in detecting significant proteomic age reduction confirms that quinoa bioester's effects are

enough to manifest in vivo, providing essential validation that complements our previous mechanistic work.

Manuscript modification: To clarify this strategic choice, we have added one sentence to the Discussion section: "For comprehensive pathway enrichment analyses of quinoa bioester's mechanisms, we refer readers to our previous work in controlled reconstructed human epidermis **(Camillo-Andrade et al., 2020)**, as the current in vivo study prioritized validating these effects in real-world human skin through our unbiased SVR approach."

9. The reduction in predicted proteomic age (11–16 years) is significant and compelling. However, the manuscript could be strengthened by discussing how such changes compare to known clinical or phenotypic measures of rejuvenation. For instance, are there benchmarks or prior studies with similar anti-aging effects (even if non-proteomic) that can serve as context?

Indeed, we consider that citing other approaches and even presenting our results with a "grain of salt", considering the limitations of our work should be emphasized. We included this contextualization in the beginning of our discussion and are happy to have had time to step back and provide additional notice. Here is our newly included opening-discussion paragraph:

"To contextualize our proteomic findings, it is important to consider established benchmarks in anti-aging research, while acknowledging that different methods of evaluating aging, such as clinical parameters, epigenetic clocks, and proteomic profiles, represent fundamentally different measurement approaches, akin to using different rulers that may not be directly comparable. That said, topical interventions have been reported to yield modest effects over weeks; for example, vitamin C formulations have shown ~10–15% improvements in skin-aging parameters (DOI: 10.3390/antiox11081605), and tretinoin, to enhance collagen remodeling (DOI: 10.1056/NEJM199308193290803). Importantly, proteomic profiling of topical cosmeceutical efficacy has rarely been performed in humans; most studies have focused on pharmaceutical actives or irritants (DOI: 10.1371/journal.pone.0097772; 10.1038/s41598-023-37750-5), with cosmeceutical ingredients typically evaluated only in cell cultures and 3D skin models (DOI: 10.1038/s41598-020-76325-6). Systemic interventions using epigenetic clocks demonstrate larger but still modest effects; for example, metformin shows approximately

2-3 years of biological age deceleration over extended periods (DOI: 10.1016/j.cell.2024.08.021).

Our observed proteomic age shifts, although substantial, must be interpreted with caution given the inherently noisy nature of proteomic data from human skin samples, which show a high inter-individual variability. We emphasize that these numbers represent a suggestive trend, and our primary contribution is demonstrating that machine learning can detect treatment-associated proteomic patterns in an unbiased way, that is, without relying on a specific marker such as collagen. In addition, the use of a paired design, where treated regions were compared against control regions within the same participant before analysis across individuals, helped reduce variability, since part of the biological 'noise' was normalized internally. Nevertheless, considering this caveat along with other limitations of the study, such as having only one participant per age, we recommend that our numerical predictions eventually be tested against larger and more diverse populations.

10. Although the study was conducted in women, and menopause is mentioned, it would be valuable to discuss whether hormonal status was assessed or controlled for, and whether the findings may translate to males or postmenopausal populations beyond age grouping. How might these findings apply to individuals with different skin types, and ethnic backgrounds?

We thank the reviewer for highlighting these important considerations regarding study generalizability. We did not assess individual hormonal status, instead using age 50 as a pragmatic cutoff to approximate pre- and post-menopausal groups, which we acknowledge as a limitation. Our study cohort was intentionally restricted to female participants with Fitzpatrick skin types II-IV from a single geographic location to minimize variability due to experimental feasibility and budget. We fully agree that extending this work to include males, diverse ethnic backgrounds, and different skin phototypes represents critical next steps, but we deliberately avoided speculating about generalizability without supporting data. We are seeking funds to expand this study.

11. The manuscript notably lacks a conclusion section, which is essential for summarizing the key findings, reinforcing the scientific contributions, and outlining the broader implications of the work.

In order to address this suggestion, we included a concluding paragraph at the end of the manuscript. We reflected it below for your convenience.

Conclusion

"*This study presents an unbiased machine learning framework using Support Vector Regression to assess whether proteomic profiles can identify younger molecular signatures in skin following cosmeceutical treatment. To our knowledge, we report the largest skin proteomic dataset to date (244 samples from 61 participants, analyzed in technical duplicates). Our data demonstrated that 30-day topical treatment with quinoa bioester induced measurable shifts in the skin proteome, with treated samples showing predicted proteomic ages younger than placebo-treated controls, particularly in participants aged 50 and above. The paired proteomic analysis revealed coordinated changes in proteins governing skin barrier function (DSG1, DSC1, FLG), oxidative defense (SOD1, GLRX, GSS), and protease regulation (SERPINB3, SERPINB7), consistent with mechanisms of skin rejuvenation. While our findings are limited to female participants with Fitzpatrick skin types II-IV and require validation in more diverse populations, this work establishes a quantitative framework for evaluating anti-aging interventions that moves beyond subjective assessments. Future studies should explore treatment persistence, dose-response relationships, and extend to broader demographic groups. Despite inherent challenges in modeling complex biological aging processes, this proof-of-concept demonstrates that machine learning approaches can detect treatment-induced proteomic shifts in human skin, providing a foundation for objective assessment of cosmeceutical efficacy and opening new avenues for evidence-based and unbiased evaluation of skin health interventions.*"

12. A few additional experiments or analyses could further strengthen the scientific rigor and impact of the study, particularly given the standards of Communications Biology. (i) Exploring different concentrations of quinoa bioester or sampling at multiple time points during treatment could reveal the dynamics and optimal conditions of its effect on skin proteomics. (ii) Conducting a systems-level analysis (e.g., gene ontology or pathway enrichment analysis) of the differentially abundant proteins would provide mechanistic insights and strengthen the biological interpretation of the findings.

While dose-response and temporal dynamics studies would indeed be valuable, implementing these would require an entirely new clinical trial. Our current study involved 61 participants and we took over 4 years with 244 proteomic samples, representing substantial investment of resources and participant commitment. Adding concentration variations and multiple timepoints would multiply this complexity several-fold, plus requiring funding for personnel of which we don't currently dispose.

As noted in our response to point 8, we previously conducted comprehensive pathway enrichment analyses using quinoa bioester in controlled reconstructed human epidermis (Camillo-Andrade et al., Scientific Reports, 2020). The current study was specifically designed to validate whether these mechanistic effects translate to real-world human skin. Repeating GO analysis on this inherently noisier in vivo dataset would yield less

interpretable results than our previous controlled study, which readers can reference for detailed mechanistic insights.

13. The manuscript does not exhibit any major technical or conceptual flaws that would prohibit its publication. The study is methodologically sound and presents an innovative approach using support vector regression (SVR) on proteomic data to estimate biological skin age and evaluate the rejuvenating potential of quinoa bioester.

We are greatly encouraged by Reviewer #4's concluding remarks that the manuscript is "methodologically sound" and does not exhibit "any major technical or conceptual flaws" is deeply appreciated. We thank the reviewer once again. We firmly believe that the revisions undertaken have further strengthened the work.

Dear Dr. Dario Ummarino,

We thank the you and Reviewer #2 for the final comments on how to get our manuscript ready for the publication format and for the opportunity to revise our manuscript. We have carefully addressed each point raised below. Our responses are provided beneath each comment.

Editor's requests

1. Please ensure that the classification of your study as an exploratory, proof-of-concept, prospective observational study is clearly specified in the abstract, introduction and discussion sections. References to 'efficacy study' should be removed throughout the manuscript. Please also ensure the main pre-specified outcome of your study is clearly reported throughout the manuscript. ***Note: We have consolidated this point with the separate abstract-specific request ("Please remove reference to 'randomized efficacy study', and define the study in line with the original study design"), as both address the same concern regarding study classification terminology. Our response below covers both.***

We revised the manuscript to ensure the study is consistently classified as an "exploratory, proof-of-concept, prospective observational study" in the abstract, introduction, and discussion sections. All references to "efficacy study" have been removed, and the pre-specified primary outcome is now clearly stated. The specific changes are detailed below.

Abstract

Before: "We conducted a randomized, vehicle-controlled cosmetic efficacy study, where 61 women (ages 20-80) received 30-day topical quinoa bioester treatment."

After: "In this exploratory, proof-of-concept, prospective observational study, we set out to characterize treatment-induced proteomic changes in human skin as the pre-specified primary outcome. Sixty-one women (ages 20-80) received 30-day topical quinoa bioester application on one forearm, with the contralateral forearm receiving vehicle."

Introduction

Before: "In this work, we employed a differential proteomic pipeline using paired sampling from the forearms of 61 individuals..."

After: "Here, we employed a differential proteomic pipeline using paired sampling from the forearms of 61 individuals..."

Before: "These properties underscore its potential efficacy in decelerating the aging process at the molecular level"

After: "These properties underscore its potential for alterations at the molecular level"

Discussion

Before: "proteomic profiling of topical cosmeceutical efficacy has rarely been performed in humans"

After: "proteomic profiling of topical cosmeceutical effects has rarely been performed in humans"

Conclusion (now merged into Results and Discussion)

Before: "This study presents an unbiased machine learning framework using Support Vector Regression to assess whether proteomic profiles can identify younger molecular signatures in skin following cosmeceutical treatment."

After: "This observational study presents an unbiased machine learning framework using Support Vector Regression to assess whether proteomic profiles can detect shifts in molecular signatures of skin following cosmeceutical application."

Before: "providing a foundation for objective assessment of cosmeceutical efficacy and opening new avenues for evidence-based and unbiased evaluation of skin care formulations"

After: "providing a foundation for objective assessment of cosmeceutical effects and opening new avenues for evidence-based and unbiased evaluation of skin care formulations"

2. Please also ensure that the original study protocol is referenced in the main manuscript. Please also discuss any deviations between the study protocol and the current study. For instance, please clarify whether the Support Vector Regression analysis was included in the original protocol.

The original study protocol, approved by the ethics committee (CAAE 38352020.8.0000.5248, Parecer 4.971.427), has been included as Supplementary Note 1 (original Portuguese version and English translation) and is now referenced in the Methods section. The specific changes are detailed below.

Regarding deviations: the study was conducted in full accordance with the approved protocol. No deviations occurred in study population, sample collection, treatment

procedures, or data acquisition. The specific machine learning approach (Support Vector Regression) was not defined in the original protocol. This is consistent with standard practice in the Brazilian CEP/CONEP ethics review system, where study protocols specify the research design, data collection procedures, and primary analytical objectives, while nuances in the computational methodology are determined during the analytical phase. We even recall that in a past revision, one of the reviewers suggested us of using the LASSO, to which we argued that it indeed was our first choice and that we experimented with it. The SVR-based proteomic age prediction emerged, in our hands, as the most suitable approach for evaluating treatment-associated proteomic shifts during data analysis.

Methods, Sample Collection

Before: "This study received approval from the ethics committee (CAAE 38352020.8.0000.5248)."

After: "This study received approval from the ethics committee (CAAE 38352020.8.0000.5248). The original approved study protocol is provided in Supplementary Note 1."

Methods, SVR section

Before: "We aim to predict a proteomic age based on skin proteomic profiles; for this, we used Python 3.8 with widely adopted libraries such as pandas for data handling, NumPy for numerical computations, scikit-learn for machine learning, and matplotlib for graphical visualizations."

After: "We aim to predict a proteomic age based on skin proteomic profiles. The SVR-based approach was later selected during the analytical phase as the most suitable method for evaluating proteomic age shifts, consistent with the exploratory nature of this study. For this, we used Python 3.8 with widely adopted libraries such as pandas for data handling, NumPy for numerical computations, scikit-learn for machine learning, and matplotlib for graphical visualizations."

3. Please provide the details of the participants' randomization procedure in the methods section.

The details of the participants' allocation procedure have been added to the Methods section. Upon reviewing the procedure for this revision, we recognized that the forearm assignment followed a systematic balanced allocation rule (odd-aged participants received the quinoa bioester formulation on one forearm, even-aged participants on the other) rather than a randomized procedure. We have accordingly corrected the terminology throughout the manuscript to accurately reflect this. The allocation achieved

balanced distribution across forearms and, combined with participant blinding, controlled for potential lateral dominance effects. All instances of "randomized" referring to treatment allocation have been updated as detailed below.

Results, subsection heading

Before: "Bilateral Forearm Sampling and Randomized Product Assignment to Minimize Experimental Bias and Enable Paired Analysis."

After: "Bilateral Forearm Sampling and Systematic Product Allocation to Minimize Experimental Bias and Enable Paired Analysis."

Results, subsection text

Before: "Aiming toward establishing a well-controlled evaluation of quinoa bioester effects, we employed a bilateral forearm sampling strategy with randomized treatment allocation."

After: "Aiming toward establishing a well-controlled evaluation of quinoa bioester effects, we employed a bilateral forearm sampling strategy with systematic balanced treatment allocation."

Before: "Our motivation in randomizing treatment assignment between forearms was to help minimize potential biases introduced by lateral dominance, ensuring that any observed differences could be attributed to the treatment rather than systematic physiological asymmetries."

After: "Our motivation in alternating treatment assignment between forearms was to help minimize potential biases introduced by lateral dominance, ensuring that any observed differences could be attributed to the treatment rather than physiological asymmetries."

Methods, Sample Collection

Before: "The assignment of which forearm (left or right) received the quinoa bioester-containing topical formulation or the vehicle was randomized among participants to control for potential side dominance effects. Formulation tubes were labeled 'right forearm' or 'left forearm' with accompanying written instructions to ensure correct application throughout the treatment period. Both formulations were identical in appearance and texture, and participants were blinded to which formulation contained the active ingredient."

After: "Formulation allocation followed a systematic balanced rule: odd-aged participants received the quinoa bioester formulation on one forearm and the vehicle on the contralateral forearm, while even-aged participants received the reverse assignment. This ensured balanced distribution of the active formulation across left and right forearms. Formulation tubes were labeled 'right forearm' or 'left forearm' with accompanying written

instructions to ensure correct application throughout the treatment period. Both formulations were identical in appearance and texture, and participants were blinded to which formulation contained the active ingredient."

4. We request that the phrase 'Younger Molecular Signatures' is changed for scientific accuracy. Please also write the title in lowercase.

The title has been revised for scientific accuracy and reformatted in lowercase per journal style.

Title

Before: "Quinoa Bioester Application Shifts Human Skin Proteome Toward Younger Molecular Signatures"

After: "Quinoa bioester application shifts human skin proteome toward molecular profiles associated with younger age"

Note: the only other occurrence of the phrase "younger molecular signatures" in the manuscript (in the conclusion) was already revised as part of our response to point 1.

5. Please remove the 'Conclusions' heading and move its content to the end of the 'Results and Discussion' section.

The "Conclusion" heading has been removed and its content has been relocated to the end of the "Results and Discussion" section.

6. Please ensure to provide an 'Author Contributions' section that individually lists the specific contribution of each author to the work. Each author must be referred to by name or initials. Where multiple authors possess identical initials, they must be clearly disambiguated from one another.

An Author Contributions section has been added to the manuscript as reflected below:

Author Contributions

ACCA performed skin sample collection, proteomic sample preparation, data analysis, and co-wrote the manuscript. LAS developed the SVR pipeline, wrote the computational code, and co-wrote the manuscript. CMC, BB, CFSO, PHS, ADPMC, ARIF, and RCR contributed to formulation preparation, experimental design, and project conception. GD and DCS contributed to formulation preparation, experimental design, project conception, and supervised the research at Grupo Boticario. RD and MDMS contributed to proteomic data analysis. MDMS also contributed to code development and sample preparation.

JSGF contributed to sample preparation and co-supervised ACCA. PCC conceived and supervised the project, contributed to the SVR approach design, and co-wrote the manuscript. All authors reviewed and approved the final manuscript.

7. Please ensure to provide a 'Competing interests' statement using one of the standard sentences specified by the journal.

The heading has been changed from "Conflict of interests" to "Competing interests" per journal style. Minor errors in author initials were corrected.

8. The Methods should include a separate section titled "Statistics and Reproducibility" with general information on how the statistical analyses of the data were conducted, and general information on the reproducibility of experiments, including the sample sizes and number of replicates and how replicates were defined.

We included the following new section:

Statistics and Reproducibility

This study enrolled female participants (one per age, 20 to 80 years), each providing four skin samples (two forearms, before and after treatment), totaling 244 biological samples. All samples were analyzed by mass spectrometry in technical duplicates (488 raw files). Technical replicates were defined as repeated LC-MS/MS acquisitions of the same peptide preparation; biological replicates were defined as samples from different participants. Quality control and LC-MS-MS reproducibility of the mass spectrometry data was performed using RawVegetable[1], and a detailed assessment is available in Supplementary File 1. No data were excluded from the analyses.

For differential proteomics, a paired one-sample t-test on log2 fold changes with Benjamini-Hochberg FDR correction (adjusted $p < 0.05$) was used, as detailed in the Paired Proteomic Analysis section. For comparisons of predicted proteomic age between treatments within the same individuals, the Wilcoxon signed-rank test was used. For comparisons between age groups, the Mann-Whitney U test was used. Skin hydration differences were assessed using a two-sided t-test. All reported p-values are two-sided.

9. Where human participants are involved, you must provide the following in the Methods section: a statement confirming that informed consent was obtained; the name of the board and institution that approved the study protocol; and the

following statement: "All ethical regulations relevant to human research participants were followed."

The ethics statement in the Methods section has been revised to include all three required elements. The specific change is detailed below.

Methods, Sample Collection

Before: "This study received approval from the ethics committee (CAAE 38352020.8.0000.5248). Our inclusion criteria considered only female individuals aged between 20 and 80 years with phototypes II to IV. Exclusion criteria encompassed conditions such as skin diseases, smoking, diabetes and pregnancy. Age-stratified enrollment. We prospectively used single-year quota sampling across the prespecified 20-80 range. Volunteers were openly recruited and screened with a standardized form, ID verification, and considering year of birth; we enrolled one participant per birth-year. Age was defined as completed years at baseline, ensuring one participant of each age. All methods were performed in accordance with relevant guidelines and regulations, with written informed consent obtained from all participants."

After: "This study was approved by the Research Ethics Committee of the Instituto Oswaldo Cruz, Fundacao Oswaldo Cruz (CEP FIOCRUZ/IOC; CAAE 38352020.8.0000.5248, Parecer 4.971.427). All ethical regulations relevant to human research participants were followed. Written informed consent was obtained from all participants. Our inclusion criteria considered only female individuals aged between 20 and 80 years with phototypes II to IV. Exclusion criteria encompassed conditions such as skin diseases, smoking, diabetes and pregnancy. Age-stratified enrollment. We prospectively used single-year quota sampling across the prespecified 20-80 range. Volunteers were openly recruited and screened with a standardized form, ID verification, and considering year of birth; we enrolled one participant per birth-year. Age was defined as completed years at baseline, ensuring one participant of each age."

10. It is mandatory to provide access to the numerical source data for graphs and charts, either deposited in a repository or uploaded as Supplementary Data.

The numerical source data underlying Figures 3 and 4 are now provided as Supplementary Data 1 (Excel format).

Data Availability

Before: “The mass spectrometry proteomics data have been deposited to the ProteomeXchange Consortium via the PRIDE partner repository with the dataset identifier PXD062216.”

After: “The mass spectrometry proteomics data have been deposited to the ProteomeXchange Consortium via the PRIDE partner repository with the dataset identifier

PXD062216. Source data underlying the graphs in Figures 3 and 4 are provided in Supplementary Data 1. Quality control assessments are available in Supplementary File 1, protein identifications in Supplementary File 2, differential proteomics results in Supplementary File 3, and skin hydration measurements in Supplementary File 4. All other data supporting the findings of this study are available from the corresponding authors upon reasonable request”

11. Please ensure the PXD062216 is publicly released prior to resubmission.

The mass spectrometry proteomics dataset (PXD062216) has been made publicly available on the PRIDE repository prior to this resubmission.

12. Please ensure that the version of the code/software described in the paper is deposited in a DOI-minting repository (e.g., Zenodo) and that this DOI is also cited in the main Reference list.

The code available at https://github.com/marlondms/AI-ON-SKIN/ has been archived on Zenodo via the GitHub-Zenodo integration and assigned a persistent DOI. The DOI has been cited in the main reference list and referenced in the Methods section.

Methods, SVR section

Before: "Our code is available at https://github.com/marlondms/AI-ON-SKIN/."

After: "Our code is available at https://github.com/marlondms/AI-ON-SKIN/ and has been archived on Zenodo (DOI: 10.5281/zenodo.19042083)."

References

The following reference was added: Santos, M. D. M. AI-ON-SKIN: SVR-based proteomic age prediction pipeline [Software]. *Zenodo* https://doi.org/10.5281/zenodo.19042083 (2026).

13. The Data Availability statement must include access details for deposited data, including repository name and unique data ID, and how source data can be obtained.

The Data Availability statement has been expanded to include access details for all deposited data and source data. A separate Code Availability section has been added as required. The specific changes are detailed below.

Data Availability

Before: "The mass spectrometry proteomics data have been deposited to the ProteomeXchange Consortium via the PRIDE partner repository with the dataset identifier PXD062216."

After: "The mass spectrometry proteomics data have been deposited to the ProteomeXchange Consortium via the PRIDE partner repository with the dataset identifier PXD062216. Source data underlying the graphs in Figures 3 and 4 are provided in Supplementary Data 1. Quality control assessments are available in Supplementary File 1, protein identifications in Supplementary File 2, differential proteomics results in Supplementary File 3, and skin hydration measurements in Supplementary File 4. All other data supporting the findings of this study are available from the corresponding authors upon reasonable request."

Code Availability (new section, added after Data Availability)

"The SVR-based proteomic age prediction pipeline is available at https://github.com/marlondms/AI-ON-SKIN/ and has been archived on Zenodo (DOI: 10.5281/zenodo.19042083). The pipeline was implemented in Python 3.8 using scikit-learn, pandas, NumPy, and matplotlib."

14. Please define the error bars in each Figure and Supplementary Figure where they are used.

Our only Figure that has error bars is Figure 4; the caption has been updated to define the boxplot elements, as detailed below.

Figure 4 caption

The following sentence was added at the end of the caption: "In each boxplot, the center line represents the median, box edges represent the first and third quartiles (Q1 and Q3), and whiskers extend to the most extreme data points within 1.5 times the interquartile range (IQR) from the box edges. Points beyond the whiskers represent outliers."

15. Each Figure must be provided as a separate file. Figure captions must be included within the main manuscript file, grouped together at the end of the document.

All figures have been removed from the main manuscript file and are now provided as separate files (Fig1.tiff, Fig2.tiff, Fig3.tiff, Fig4.tiff) at 300 dpi minimum resolution. Placeholders indicating figure placement (e.g., "[Figure 1 about here]") have been inserted at the original positions in the text. A new "Figure Captions" section has been

added at the end of the main manuscript file, after the references, containing all figure captions grouped together.

16. "Homepage summary (as requested in the Final Revision Instructions checklist):

Paired skin proteomic comparisons within the same individuals reveal quinoa bioester application modulates barrier and antioxidant proteins, shifting molecular profiles toward those associated with younger age, with pronounced effects after age 50."

Reviewer #2

The concerns raised by us previously, have mostly been addressed. The goal of the study and hence the chosen methodology is now more understandable. However, we feel that stating the 11-16 years reduction in proteomic age is not established. The authors point to the domain shift causing the post-treatment prediction shifting upwards compared to the untreated predictions, and because of this the absolute age estimations are not reliable. The authors state that "our primary contribution is demonstrating differential treatment effects rather than precise absolute age estimation in treated skin", which we highly support. In order to align with this, we suggest refining the conclusions on exact age reduction.

We agree with Reviewer #2 and have revised the manuscript to make even more evident that the observed numerical differences in predicted proteomic age (11 and 16 years for the under-50 and over-50 groups, respectively) do not necessarily reflect biological years and should be interpreted as directional indicators of a differential treatment effect rather than calibrated absolute age changes. All changes are detailed below.

Abstract

Before: "predicted quinoa bioester-treated skin as 11-16 years younger than vehicle-treated skin ($p<0.01$ for participants ≥50 years). This represents the first proteomic age clock for human skin and demonstrates that topical bioactives can shift molecular signatures toward younger states."

After: "predicted lower proteomic ages for quinoa bioester-treated skin compared to vehicle-treated skin, with observed median differences of 11 and 16 years for participants under and over 50, respectively ($p<0.01$ for participants ≥50 years). While these values do not necessarily correspond to biological years, these findings demonstrate that topical bioactives can induce detectable shifts in skin proteomic profiles."

Figure 4 caption

Before: "p=0.14 for the <50 group (median reduction of 11 years) and p<0.01 for the ≥50 group (median reduction of 16 years), comparing bioactive to vehicle within each age stratum."

After: "p=0.14 for the <50 group (observed median difference of 11 years) and p<0.01 for the ≥50 group (observed median difference of 16 years), comparing bioactive to vehicle within each age stratum. These values demonstrate differential treatment effects and do not necessarily correspond to biological years."

Results, treatment effect paragraph

Before: "The magnitude of the treatment effect showed an age dependence, with the older cohort experiencing a more substantial reduction in predicted proteomic age. While younger participants showed a median reduction of 11 years compared to vehicle (p = 0.14, Wilcoxon signed-rank test), older participants demonstrated a 16-year reduction (p < 0.01, Wilcoxon signed-rank test), suggesting that quinoa bioester may be particularly effective in addressing the molecular patterns of skin aging."

After: "The magnitude of the differential treatment effect showed an age dependence. Younger participants showed an observed median difference of 11 years between treatments (p = 0.14, Wilcoxon signed-rank test), while older participants showed an observed median difference of 16 years (p < 0.01, Wilcoxon signed-rank test). These numerical differences do not necessarily reflect biological years due to inherent limitations of applying the model to post-treatment data (domain shift), but indicate a differential effect that is more pronounced in older skin."

Results, consistency paragraph

Before: "The consistency of these findings across both age cohorts, coupled with the more pronounced effects in older participants, strongly suggests toward the rejuvenating potential of quinoa bioester."

After: "The consistency of these findings across both age cohorts, coupled with the more pronounced effects in older participants, supports a directional proteomic effect of quinoa bioester relative to vehicle."[

Before: "The proteomic age reduction observed with quinoa bioester treatment was supported by quantitative hydration measurements"

After: "The proteomic age difference observed between quinoa bioester and vehicle treatments was supported by quantitative hydration measurements"

Discussion, pathway paragraph

Before: "may explain the substantial age reduction observed in our model."

After: "may explain the pronounced differential effect between treatments observed in our model."

Discussion, caution paragraph

Before: "Our observed proteomic age shifts, although substantial, must be interpreted with caution"

After: "Our observed differences in predicted proteomic age between treatments must be interpreted with caution"

We hope these revisions adequately address the concerns raised. We remain available for any further clarification.

Sincerely, Paulo C. Carvalho, on behalf of all authors
